# Comprehensive Scoping Review on Body Image Perceptions and Influences in Children and Adolescents

**DOI:** 10.3390/ejihpe14100179

**Published:** 2024-10-04

**Authors:** Suellem Zanlorenci, Leticia Gonçalves, Mikael Seabra Moraes, Leandro Narciso Santiago, Matheus Silveira Pedroso, Diego Augusto Santos Silva

**Affiliations:** Department of Physical Education, Sports Center, Federal University of Santa Catarina, Florianopolis 88040900, Brazil; suellemzan@gmail.com (S.Z.); leticia.g.2008@hotmail.com (L.G.); moraesmikael@gmail.com (M.S.M.); santiago.imagine@gmail.com (L.N.S.); matheus.pedroso1994@gmail.com (M.S.P.)

**Keywords:** body image, children, adolescents, mental health, PRISMA review, scoping review, health promotion

## Abstract

Conducting a scoping review helps identify research gaps and opportunities, avoid duplication, guide the selection of appropriate methodologies, and base studies on existing evidence. The aim of this study was to map the literature on body image in children and adolescents (0 to 19 years). The present study follows the recommendations of the Preferred Reporting Items for Systematic Reviews and Meta-Analyses Extension for Scoping Reviews (PRISMA). The search was conducted in the following databases: PubMed, Web of Science, Scopus, SPORTDiscus, LILACS, SciELO, PsycINFO, CINAHL, and the Cochrane Library. A total of 3257 articles were found, of which 2147 were duplicates, resulting in 1110 articles. Of these, 41 met the inclusion criteria. The results were divided into analytical dimensions, including measurement instruments, programs and interventions, social media, sociodemographic aspects, physical activity, personality and cognitive thinking, and studies with specific populations. The results highlight that peer influence, physical activity, media, and the school environment play crucial roles in shaping young people’s body image; factors such as sex, age, and socioeconomic context emerge as important variables in understanding body perceptions, and educational interventions and health promotion programs have been shown to be effective in preventing and reducing body dissatisfaction, underscoring the need for multifactorial and collaborative approaches.

## 1. Introduction

Body image is defined by how people experience their own body. More broadly, body image can be related to functional physical competencies and/or biological integrity [1]. Therefore, there are various constructs and perspectives on body image; for example, the tridimensional model of body image, which comprises perception (how the body is seen), attitude (how the feelings about the body are), and behavior (what actions are taken in relation to the body) [1]. Another example is the subjective theory, which encompasses sociocultural aspects (demographic aspects, media, and excessive exposure to social media) and developmental aspects (weight control behaviors, provocative behaviors characterized by bullying, hormonal changes, and rapid growth caused by the puberty period) [2].

Although present at all stages of life, body image dissatisfaction can develop during childhood, as children in this period may exhibit concerns about physical appearance [3]. However, it can also be present during adolescence [4], as adolescents strive to meet the beauty standards and expectations of the society in which they live [4]. Body image dissatisfaction in children and adolescents can vary according to various factors, including age, sex, culture, socioeconomic context, and even media and societal influences [5]. A study conducted in 2017 in Malaysia with 776 school adolescents aged 11 to 12 years estimated that 60.1% of the sample was dissatisfied with their body image [5]. Factors associated with body image dissatisfaction among Malaysian schoolchildren included being overweight/obese and being female [5]. Research conducted between 2013 and 2014 with 18-to-19-year-old adolescents in Brazil estimated that 66.5% were female, 32.8% were overweight, and 11.99% were obese [6]. Another finding of the same research was the association of body dissatisfaction due to overweight with symptoms of depressive disorder, while there was no evidence of body image dissatisfaction related to thinness [6].

In 2018, about 90% of adolescents used social media daily [7]. The proliferation of images focusing on physical appearance on social media can further promote body dissatisfaction [8]. The central reason for this is that idealized images often emphasize body appearances [9], meaning that seeing and following influencers and celebrities on social media can increase social comparisons and intentions to change one’s appearance, particularly concerning fitness and diet [10]. In some cases, family and parental issues, such as feelings of injustice, loneliness, and parental neglect, are determining factors for body dissatisfaction [11]. Numerous negative health consequences occur in children and adolescents who are dissatisfied with their body image, such as depressive symptoms, increased anxiety attacks, low self-esteem, harmful behaviors for weight control, and the development of eating disorders [12,13].

Conducting a scoping review helps identify research gaps and opportunities, avoid duplication, guide the selection of appropriate methodologies, and base studies on existing evidence [14]. Performing a scoping review also contributes to positioning the work in relation to current knowledge for scientific advancement within a specific field of study, synthesizing the state of the art on a particular topic [14]. In this sense, considering the broad scope of the topic of body image in children and adolescents, it was thought that a scoping review of systematic reviews could be the starting point for compiling existing evidence and knowledge gaps regarding body image in children and adolescents into a single document. Thus, the objective of the present study was to map the literature on body image, bringing together the evidence on this topic in children (zero to nine years old) and adolescents (10 to 19 years old).

## 2. Method

The scoping review methodology was chosen, which is a method frequently used to recognize the literature on a specific topic; assist in mapping studies; analyze the extent, scope, and nature of the investigation; summarize and disseminate research data; and identify existing research gaps [15,16]. A scoping review possesses the transparency and replicability provided by the stages of systematic review, without the purpose of evaluating the quality of the produced evidence [17].

The reporting of this review followed the recommendations of the *Preferred Reporting Items for Systematic Reviews and Meta-Analyses extension for Scoping Reviews* (PRISMA-ScR) [18].

### 2.1. Search Strategy, Descriptors, and Keywords

The search was conducted in the following databases: (1) PubMed via the National Library of Medicine (MEDLINE); (2) Web of Science; (3) Scopus; (4) SPORTDiscus via EBSCOhost; (5) LILACS via the Virtual Health Library; (6) Scientific Electronic Library Online (SciELO); (7) PsycINFO via the American Psychological Association (APA); (8) Cumulative Index to Nursing and Allied Health Literature (CINAHL), via EBSCOhost; and (9) the Cochrane Library.

The search for articles in the databases was performed using the advanced search tool, based on the construction of blocks of descriptors and keywords related to the topic. The selection of descriptors was carried out by consulting the Medical Subject Headings (MeSH) platforms. Depending on the database, keywords and descriptors were entered in Portuguese, English, and/or Spanish.

The first block (outcome) comprised terms related to body image, the second block comprised the population of interest (children and adolescents), and the third block comprised terms related to systematic reviews and meta-analyses (Supplementary Material).

The Boolean operator “OR” was used to add at least one keyword or descriptor from each block in the advanced search, and the operator “AND” was used to relate the blocks of keywords/descriptors to each other. Additionally, quotation marks (“”) were used for compound words and to search for exact terms or expressions. Parentheses were used to combine the search terms by categories of outcome, exposure, and population. The asterisk (*) was used to search for all words derived from the same prefix.

The search was conducted in October 2023, considering all articles published up to that month.

### 2.2. Eligibility Criteria

Systematic reviews and meta-analyses on body image in children and adolescents (0 to 19 years old) were considered eligible. Systematic review articles and meta-analyses that verified body image assessment methods in children and adolescents (0 to 19 years old). The age range up to 19 years was chosen because, according to the World Health Organization, this is the age limit for adolescents [19].

The following exclusion criterion was considered: studies that were not systematic reviews or meta-analyses on body image in children and adolescents (0 to 19 years old).

### 2.3. Study Selection and Data Extraction

Two reviewers (SZ and LG) independently examined each database to obtain potential articles. After extracting the articles from the databases, duplicates were excluded, followed by the reading and exclusion of articles based on titles and abstracts. The publications found were exported to the Rayyan application [20]. After removing duplicates, the two authors independently read the titles and abstracts of the articles and excluded those that did not meet the eligibility criteria. Subsequently, the full texts of the selected articles were read in their entirety for the final selection of studies. Additionally, a literature search was conducted in the references of the selected studies to identify potential eligible studies for this scoping review that were not identified in the systematic search of the databases. Discrepancies between the two reviewers were resolved through a consensus meeting. The opinion of a third reviewer (DASS) was sought for unresolved discrepancies.

The bibliographic manager Zotero^®^ version 5.0 (Roy Rosenzweig Center for History and New Media, Fairfax, VA, USA) was used to create specific libraries, which enabled the identification and exclusion of duplicate studies, as well as the division and organization of results from each database. Data were extracted by two independent reviewers (SZ and LNS) and consistency between them was verified by a third reviewer (DASS). The following information was extracted: names of authors, year of publication, objective of the study, number of studies included in the reviews, number of databases used for the search and the specific databases used, types of studies considered, whether or not meta-analysis was performed, total number of subjects, main results, and tests used to assess body image.

The objectives of the systematic review and meta-analysis articles included in this scoping review were analyzed by two independent reviewers (SZ and DASS) and, after consensus, these studies were grouped into the following analytical dimensions: “Body Image Measurement Instruments” (articles whose main objective was to measure body image), “Programs and Interventions Focusing on Body Image” (articles on intervention), “Sociocultural influences on body image” (articles on body image and social media use, peers, and family), “Sociodemographic Aspects Related to Body Image” (articles on body image and sociodemographic aspects), “Physical Activity and Body Image” (articles about body image and physical activity), “Personality and Cognitive Thinking for Understanding Body Image” (articles on body image, personality, and cognitive thinking), and “Studies on Body Image with Specific Populations” (articles on body image and specific populations such as patients with cancer, lupus, HIV, and chronic diseases, and patients pre- and postpartum).

The dimension “Body Image Measurement Instruments” is important because, considering that disorders related to body image affect an individual’s health, it is essential to recognize the different protocols (methods or instruments) employed for the accurate assessment of body image. Detailed knowledge of these protocols promotes the standardization and comparability of results between studies, fostering continuous advancement in research and clinical practice in this field [21]. The dimension “Programs and Interventions Focusing on Body Image” is important because researchers and professionals can identify successful strategies, adjust approaches as needed, and implement evidence-based practices to improve health and well-being outcomes related to body image [22,23]. The dimension “Sociocultural influences on body image” is important because the expectation of achieving the “ideal body”, promoted by the media, peers, and family intensifies body image dissatisfaction [24]. The dimension “Sociodemographic Aspects Related to Body Image” is important because there is significant evidence of the relevance of sociodemographic aspects in shaping feelings of body satisfaction/dissatisfaction [25,26]. This suggests that cultural influences and beauty standards can manifest in different ways, varying, for example, according to socioeconomic class [25,26].

The dimension “Physical Activity and Body Image” is important due to the impact that physical activity can have on physical, mental, and social health [27]. Regular physical activity is associated with a range of physical and mental health benefits, including improved body composition, increased self-esteem, and a reduced risk of developing chronic diseases such as obesity and cardiovascular diseases [27]. Understanding how physical activity influences perception and satisfaction with body image allows for the identification of risk and protective factors, contributing to the development of more comprehensive and personalized public health strategies aimed at improving the quality of life and well-being of children and adolescents [28]. The dimension “Personality and Cognitive Thinking for Understanding Body Image” is important because it can help improve an individual’s perception of their body, from the perspective of Cognitive Behavioral Therapy [29]. The dimension “Studies on Body Image with Specific Populations” helps to understand body image perception in specific population groups (with diagnosed disorders and/or specific diseases, as well as temporary physical conditions). In these groups, it is important to investigate the evidence on body image because this has the potential to provide insights for the development of more comprehensive and personalized public health strategies aimed at improving quality of life and well-being in these individuals [12,30,31,32,33,34,35,36,37,38].

### 2.4. Risk of Bias/Methodological Quality

The assessment of the risk of bias/methodological quality of the included systematic review studies was conducted independently by two researchers (SZ and MSM). In cases of disagreement between the two researchers, a third researcher (DASS) with experience in scoping reviews was consulted through a consensus meeting. For the risk of bias assessment, the tool proposed by the National Heart, Lung, and Blood Institute (NIH) was used according to each type of study. The Quality Assessment of Systematic Reviews and Meta-Analyses (https://www.nhlbi.nih.gov/health-topics/study-quality-assessment-tools, accessed on 14 January 2024) was employed, which is the recommended tool to assist in assessing the internal validity (potential risk of selection, information, measurement, or confounding factors) of systematic review and meta-analysis studies. The instrument consists of eight criteria that help identify potential risks of bias regarding the research problem and the use of explicit and reproducible criteria in relation to the studies included in each review [39].

Each question was scored with “0” or “1”, with “0” applied to questions answered with “no” and “1” for those answered with “yes” or “not applicable”. The “not applicable” option was used when it was not possible to evaluate one of the criteria of the instrument due to the type of study. The total score was obtained by summing the scores of each question [39].

## 3. Results

### 3.1. Study Selection

**Figure 1:**
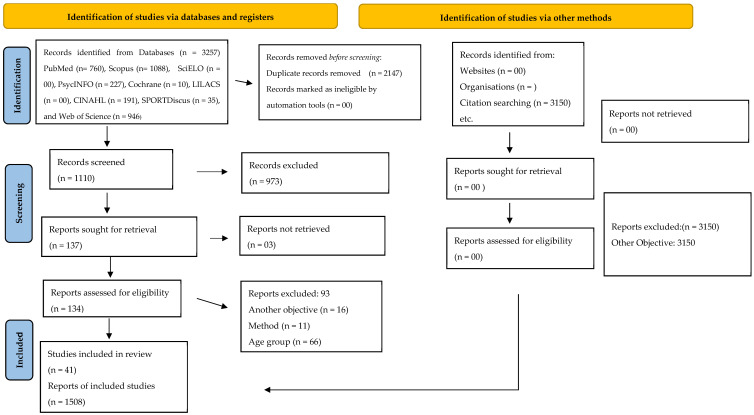
Flowchart of the search, selection, and exclusion of articles. Source: Prepared by the author (2024).

A total of 3257 articles were identified; however, 2147 were duplicates, resulting in 1110 articles. After screening the titles and abstracts, 973 studies were excluded, and subsequently, 137 articles were read in full. In total, 41 articles met the inclusion criteria of the present study. Additionally, the references of the included articles were reviewed, and no other reviews meeting the inclusion criteria of the present study were identified (Figure 1).

### 3.2. Characteristics of the Studies

Detailed information (title, authors, year of publication, objective, city of affiliation of the first author, types of studies included, total number of included studies, total number of subjects, databases searched, main results, and analytical dimensions) of the 41 systematic review and meta-analysis articles [12,26,29,30,31,32,33,34,35,36,37,38,40,41,42,43,44,45,46,47,48,49,50,51,52,53,54,55,56,57,58,59,60,61,62,63,64,65,66,67,68] included in this scoping review can be found in Supplementary Tables S1 and S2. The objectives of the systematic reviews and meta-analyses included in this scoping review were grouped as follows: “Body Image Measurement Instruments” (one article included) [40], “Programs and Interventions Focusing on Body Image” (nine articles included) [41,42,43,44,45,46,47,48,49], “Sociocultural influences on body image” (seven articles included) [50,51,52,53,54,55,56], “Sociodemographic Aspects Related to Body Image” (six articles included) [26,57,58,59,60,61], “Physical Activity and Body Image” (six articles included) [62,63,64,65,66,67], “Personality and Cognitive Thinking for Understanding Body Image” (two articles included) [29,68], and “Studies on Body Image with Specific Populations” (10 articles included) [12,30,31,32,33,34,35,36,37,38] (Supplementary Table S1).

Of the 41 systematic review and meta-analysis articles included in the present scoping review, 7 [29,36,37,38,53,54,62] did not provide information regarding the number of individuals evaluated. Among these articles without information on the number of individuals evaluated, three articles are categorized under the analytical dimension “Studies on Body Image with Specific Populations” [36,37,38], two articles under “Sociocultural influences on body image” [53,54], one article under “Physical Activity and Body Image” [62], and one article under “Personality and Cognitive Thinking for Understanding Body Image” [29]. The systematic review articles included in this scoping review that did provide information on the number of participants evaluated encompassed a total of 682,545 individuals, of both sexes, up to 19 years of age (Supplementary Table S1).

Of the 41 systematic review articles included in the present review, 14 articles conducted systematic reviews with meta-analyses [12,31,38,40,42,44,45,47,48,52,54,57,59,62]. In the dimension “Body Image Measurement Instruments”, only one systematic review with meta-analysis was identified [40]. In the dimension “Programs and Interventions Focusing on Body Image”, four systematic reviews [41,43,46,49] and five systematic reviews with meta-analyses [42,44,45,47,48] were identified. In the dimension “Sociocultural influences on body image”, five systematic reviews [50,51,53,55,56] and two systematic reviews with meta-analyses [52,54] were identified. In the dimension “Sociodemographic Aspects Related to Body Image”, four systematic reviews [26,58,60,61] and two systematic reviews with meta-analyses [57,59] were identified. In the dimension “Physical Activity and Body Image”, five systematic reviews [63,64,65,66,67] and one systematic review with meta-analysis [62] were identified. In the dimension “Personality and Cognitive Thinking for Understanding Body Image”, only two systematic reviews [29,68] were identified. In the dimension “Studies on Body Image with Specific Populations”, seven systematic reviews [30,32,33,34,35,36,37] and three systematic reviews with meta-analyses [12,31,38] were identified (Table 1).

Regarding the year of publication of the 41 systematic review articles included in the present study, it is noted that the first systematic review addressing the topic of body image was published in 2006 and was included in the analytical dimension “Sociodemographic Aspects Related to Body Image” [59]. The year with the highest number of publications of systematic reviews on body image was 2022, with seven articles published [33,41,43,46,47,51,66], four of which belong to the dimension “Programs and Interventions Focusing on Body Image” [41,43,46,47]. The specification of the number of articles per analytical dimension each year can be found in Table 1.

### 3.3. Risk of Bias/Methodological Quality

Regarding the geographical location of the corresponding authors of the 41 systematic review articles included in this scoping review, it is noted that they were from North America [35,50,54,57,59,62,65,68], South America [26,34,37,40,55,64], the Middle East [42], East Asia [32], Southeast Asia [12,45,48], Southern Europe [51,52,53,56,60,63,67], Central Europe [33,58,66], Western Europe [30,31,36,38,41,43,47], Northern Europe [46], and Australasia [29,44,49,61]. This distribution of corresponding authors for each of the analytical dimensions investigated can be found in Table 1.

Regarding the sample of the 41 systematic review articles included in the present study, 4 studies investigated only children [44,45,60,61], 26 studies investigated only adolescents [26,29,33,35,36,37,38,40,42,46,48,49,51,52,53,55,56,58,59,62,63,64,66,67,68], and 11 studies investigated both children and adolescents [12,30,31,32,34,41,43,47,50,57,65]. In relation to the sexes of the samples investigated in the systematic reviews, 5 studies investigated only females [35,38,46,48,64], 1 investigated only males [62], 19 systematic reviews focused on both sexes [12,26,34,41,42,43,49,50,51,52,56,57,58,60,61,63,66,67,68], and 16 systematic reviews did not specify the sex of the samples [29,30,31,32,33,36,37,40,44,45,47,53,54,55,59,65]. The distribution of the investigated samples for each of the analytical dimensions can be found in Table 1.

The PsycINFO database was used by the authors of 26 systematic reviews included in this scoping review [29,30,31,33,34,35,38,41,42,43,44,46,47,49,50,51,52,54,56,57,58,59,61,62,63,68]. In general, two [12,26,35,50,54,65,66], three [42,46,51,53,58,63,68], four [31,38,40,44,47,61,67], and five [30,33,49,52,55,57,62,64] databases were the number investigated by most of the systematic reviews. Furthermore, five systematic reviews included Google Scholar as a database in their search bases [31,48,49,51,58]. Table 1 specifies the number of databases investigated for each of the analytical dimensions.

Regarding the total number of original articles mapped in the 41 reviews included in the present scoping review, a total of 1508 original studies were identified. The analytical dimension that gathered the most articles in the systematic reviews was “Studies on Body Image with Specific Populations”, which mapped 523 original articles [12,30,31,32,33,34,35,36,37,38]. On the other hand, the analytical dimension that gathered the fewest original articles was “Body Image Measurement Instruments”, which included 28 original articles [40]. Table 1 presents information on each of the analytical dimensions.

Regarding the risk of bias/methodological quality of the 41 systematic review articles, only 11 review articles received the highest score on the assessment scale, indicating a low risk of bias [31,32,34,37,44,45,46,51,53,63,66]. Of these 11 systematic review articles with a low risk of bias, three were included in the analytical dimension “Programs and Interventions Focusing on Body Image” [43,44,45], two articles were included in the dimension “Sociocultural influences on body image” [51,53], two articles were included in the dimension “Physical Activity and Body Image” [63,66], and four articles are included in the dimension “Studies on Body Image with Specific Populations” [31,32,34,37]. Still regarding the risk of bias, 25 systematic review articles obtained an average classification (between six and seven points in the final score) [12,26,29,30,33,36,38,40,41,43,47,48,49,50,52,54,55,56,57,58,60,61,62,67,68] and five systematic review articles obtained a low classification (below a score of 5 in the final score) [35,42,59,64,65] (Supplementary Table S3).

### 3.4. Evidence from the Studies

#### 3.4.1. Analytical Dimension: Body Image Measurement Instruments

The systematic review with meta-analysis included in this analytical dimension aimed to synthesize studies on body satisfaction among adolescents, focusing on the use of silhouette scales [40]. Silhouette scales, by providing standardized visual representations of different body shapes, allow for an accurate assessment of body perception and satisfaction among adolescents. They are particularly useful in identifying perceptual distortions and dissatisfaction with body image [40]. The most commonly used scales were those by Stunkard et al. [69,70], Thompson and Gray [71], Collins [72], Rand and Resnick [73], and Childress et al. [74]. Additionally, six other scales were used [75,76,77,78,79,80]. Among the five most commonly used scales [69,70,71,72,73,74], only one had a validation study in the age range of 10–18 years [71,74].

#### 3.4.2. Analytical Dimension: Programs and Interventions Focusing on Body Image

The systematic review articles included in this analytical dimension examined the effects of interventions on body image, finding evidence of improvement among female adolescents through interventions that utilized cognitive dissonance, peer support, and psychoeducation [41,42,43,44,45,46,47,48,49]. Peer support and psychoeducation interventions can improve children and adolescents’ body image by promoting acceptance of body diversity, strengthening self-esteem, and providing resilience strategies against negative social influences related to appearance [43,44,46,47]. Intervention programs measured body image [41,42,43,44,45,46,47,48,49], body satisfaction [41,43], and body self-esteem [44,48]. The results also suggest that media literacy interventions have the potential to improve and reduce body dissatisfaction, particularly those that induce cognitive dissonance. Interventions can improve with regard to evaluating the effectiveness of intervention programs [39], improving body image assessment [47], the need for more research with men [41,45], and the need for more studies on media literacy approaches that consider different cultures with different standards of ideal female and male body types [42].

#### 3.4.3. Analytical Dimension: Sociocultural Influences on Body Image

The systematic review articles included in this analytical dimension reported that body dissatisfaction is influenced by multiple variables over time [50,51,52,53,54,55,56]. The sociodemographic aspects associated with higher body image dissatisfaction identified by the systematic reviews in the present study were social pressure, media influences, peers and family, and cultural differences [50,51,52,53,54,55,56]. Social pressure, peer and family influence, and cultural differences significantly shape body image in children and adolescents by establishing beauty standards and social expectations that can impact self-esteem and the development of positive or negative body image [50,51,52,53,54,55,56].

The variables most associated with body image dissatisfaction were media influence (use of social networks, aspiring to an unrealistic body) [50,51,52,53,54,55,56]. Furthermore, the results of the systematic review articles included in this analytical dimension highlighted the relationship between exposure to media images promoting the ideal body and body image concerns in both sexes [50,51,52,53,54,55,56]. This relationship is supported by variations between body dissatisfaction and body satisfaction indices [50,51,52,53,54,55,56]. Additionally, evidence indicated that frequent use of social media is significantly related to body dissatisfaction in adolescents of both sexes [50,51,52,53,54,55,56].

#### 3.4.4. Analytical Dimension: Sociodemographic Aspects Related to Body Image

The systematic review articles included in this analytical dimension reported that body dissatisfaction is influenced by multiple variables over time [26,57,58,59,60,61]. Sociodemographic aspects related to body image refer to social and demographic characteristics that can influence an individual’s perception and satisfaction with their own body, affecting how they see and feel about their body [26,57,58,59,60,61]. The sociodemographic aspects associated with higher body image dissatisfaction identified by the systematic reviews in the present study were age, socioeconomic status, ethnicity, and sociocultural context [26,57,58,59,60,61]. Furthermore, the results of the systematic review articles included in this analytical dimension revealed that the literature is divided on whether preschool-aged females experience more body dissatisfaction than males [60,61]. Parental influence appears to be an important factor in the development of body dissatisfaction in preschool children of both sexes [60,61].

According to the systematic reviews included in this analytical dimension, adolescents of both sexes are dissatisfied with their body image [26,57,58,59]. The variables most associated with body image dissatisfaction were socioeconomic status (the higher the economic level, the greater the body image dissatisfaction). These combined factors contribute to decreased self-esteem and increased body image dissatisfaction [26,57,58,59].

#### 3.4.5. Analytical Dimension: Physical Activity and Body Image

The results of the systematic review articles included in this analytical dimension revealed that higher levels of physical activity were associated with lower body image dissatisfaction [62,63,64,65,66,67]. The studies reported that body image becomes a significant determinant of continued physical activity during adolescence. Additionally, perceived physical competence and body image become more influential factors as children grow older [62,63,64,65,66,67].

#### 3.4.6. Analytical Dimension: Personality and Cognitive Thinking for Understanding Body Image

The results of the systematic review articles included in this analytical dimension support the existence of an attentional bias toward body image-related stimuli in individuals with high levels of body dissatisfaction, in contrast to those with lower levels of related concerns [68]. Additionally, the results indicated that negative body image was associated with higher levels of neuroticism and lower levels of extroversion [29].

#### 3.4.7. Analytical Dimension: Studies on Body Image with Specific Populations

There was also a significant positive association between chronic diseases, cancer, HIV, and lupus and the risk of body dissatisfaction [12,30,31,32,33,34,37]. Similar results were suggested for the gestational and postpartum periods [35,36]. The results suggest that exclusive breastfeeding is more likely among pregnant women with a higher body image, while those with body concerns had less intention to breastfeed or start breastfeeding, and those who did start had a shorter duration [38]. Additionally, levels of body dissatisfaction varied according to age, age at onset of the disease, method for assessing body image, ethnicity, and year of publication of the article [12,30,31,32,33,34,35,36,37,38].

## 4. Discussion

The discussion of this scoping review study is structured around the main evidence according to the identified analytical dimensions, allowing for a systematic and comprehensive analysis of the different facets of the topic under study (i.e., body image), ensuring that all relevant perspectives are considered [15,16].

### 4.1. Analytical Dimension: Body Image Measurement Instruments

The most commonly used silhouette scales in adolescents according to the systematic review with meta-analysis conducted by Cortês et al. [40] included those by Stunkard et al. [69,70] and Thompson and Gray [71], who used silhouettes; Collins [72], who used silhouettes specifically for children; Rand and Resnick [73], who used self-perception and satisfaction; and Childress et al. [74], who used a tool targeted at children and adolescents between the ages of 8 and 18, with a specific section for body image that deals with body shape. The popularity of these scales suggests their acceptance and trust within the scientific community; however, most of them lack specific validation for the 10–18 age range, with the exception of the Thompson and Gray scale [71,81]. This raises concerns about the applicability and accuracy of these scales when used with adolescents, highlighting the need for more validation studies in this age group [71,81].

In addition to the five main scales, six other scales were also used in studies [75,76,77,78,79,80], indicating a diversity of tools in body satisfaction research. While this diversity can enrich studies, it also presents challenges for the standardization and comparability of results, which are essential for advancing knowledge in the field [75,78,79,80]. Future research should prioritize the validation of body satisfaction scales for different age groups to improve the quality and utility of the data obtained [34,77]. The robust validation of these scales can enhance the accuracy and relevance of body satisfaction assessments, contributing to more effective interventions and public health policies aimed at promoting positive body image across different age groups [40].

### 4.2. Analytical Dimension: Programs and Interventions Focusing on Body Image

The results highlighted the effectiveness of interventions targeted at female adolescents, particularly those employing strategies such as cognitive dissonance, peer support, and psychoeducation [41,42,43,44,45,46,47,48,49]. Interventions using cognitive dissonance have proven effective due to positive changes in dysfunctional beliefs and attitudes about the body [41]. Cognitive dissonance occurs when there is inconsistency between an individual’s attitudes and behaviors [82]. Applied to body image, this approach involves exposing adolescents to information or activities that challenge internalized beliefs about beauty ideals [82]. Through the induction of cognitive dissonance, participants are encouraged to reconsider and eventually modify their attitudes about the body, resulting in a reduced pursuit of the thin ideal and decreased body dissatisfaction [82].

Peer support can also contribute to improving body image. Positive body image is associated with better-quality interpersonal relationships during adolescence, as relationships with parents, friends, and romantic partners appear to be fundamental to the development of positive self-representations [41,83]. Psychoeducation can be beneficial for adolescents experiencing negative body image because it is easy to implement and promotes concepts of body appreciation, beauty, and self-care [41]. However, psychoeducational interventions resulted in smaller effects for male adolescents [41]. Male adolescents may face different social pressures regarding body image compared to females [84]. While female adolescents often face pressures to achieve thinness ideals, male adolescents frequently feel the pressure to attain a muscular and athletic body, an ideal often idealized by the media [84]. Thus, male adolescents may be less inclined to participate in discussions about body image [85]. This can result in lower engagement in psychoeducational interventions that rely on open discussions and personal reflections [41,85].

To make psychoeducational interventions effective for males, certain strategies can be considered: incorporating specific content that addresses and demystifies the ideals of a muscular and athletic body and their unreality, discussing the detrimental consequences for physical and mental health [86]. Interventions that promote a healthy body image should be linked to functionality and overall well-being rather than aesthetic appearance [86]. Carrying out intervention programs focused on body image and body dissatisfaction during childhood and adolescence can promote psychological, emotional, and physical well-being [41]. These interventions may help prevent eating disorders, reduce anxiety and depression, promote healthy habits, and improve academic performance and interpersonal relationships [41,42]. The studies presented in this scoping review demonstrate that planned approaches include education, psychological support, cognitive dissonance, and psychological peer support to achieve this goal.

### 4.3. Analytical Dimension: Sociocultural Influences on Body Image

The variables most associated with body image dissatisfaction were media influence (the use of social networks and aspiring to an unrealistic body), and the influence of peers and family [53,54]. For example, parental expectations regarding academic performance and extracurricular activities can exacerbate the pressure to achieve certain beauty standards [53,54]. These combined factors contribute to decreased self-esteem and increased body image dissatisfaction [53,54]. The sociocultural aspects of the subjective theory of body image can help explain the findings in this analytical dimension [5]. This theory proposes that peers, family, and the environment in which an individual is situated are essential sociocultural channels for the idealization of body image. In other words, body image is largely determined by the social experience in which the individual is embedded [24]. During adolescence, both females and males undergo significant physical and psychological changes, as this is the phase where they strive to meet beauty standards and the expectations of the society in which they live [24]. Additionally, the idealization of bodies promoted by the media, where unattainable and unrealistic beauty models are constructed, can lead adolescents who do not meet these standards to experience teasing or bullying, resulting in body image dissatisfaction [5,24,87].

The plausibility of the relationship between body image and exposure to media content has been associated with the potential effect of beauty standards propagated by social media, which idealize thin bodies for women and muscular bodies for men [83,87,88]. When these standards are not met, they can contribute to body image dissatisfaction. However, although the results are based on a large body of evidence [83,87,88], the use of social networks is, therefore, complex, since the specificities of each media exposure must be taken into account, as well as considering that this medium is an integral part of the daily lives of adolescents. In this sense, it is speculated that not all use of social media is necessarily harmful to body image [83]; this is because it could contribute to consolidating the development needs of adolescents through a social interaction network [83], as well as contributing to interventions related to body dissatisfaction, given the use of positive messages associated with the heterogeneity of different types of body image [87].

Therefore, it is hypothesized that future studies are needed to confirm the identified findings, aiming to moderate the magnitude of the associations found. This includes examining the types of social media, frequency of use, exposure to appearance-related content, and issues related to eating disorders. Additionally, given that the magnitude of the relationship between exposure to media images tends to intensify during adolescence, possibly due to physiological and anatomical transformations related to puberty [87], the consideration of age in the included studies can significantly contribute to determining body dissatisfaction. Thus, in addition to the need for a greater body of evidence from longitudinal studies in different age groups to confirm the results of this review, it is suggested that future studies consider different age groups, since the associations and conclusions identified may vary depending on the inclusion of this information.

### 4.4. Analytical Dimension: Sociodemographic Aspects Related to Body Image

According to the systematic reviews included in this analytical dimension, children and adolescents of both sexes are dissatisfied with their body image [26,57,58,59,60,61]. The variables most associated with body image dissatisfaction were socioeconomic status (the higher the economic level, the greater the body image dissatisfaction). Individuals from higher economic classes are often exposed to social environments where the emphasis on physical appearance is predominant [26]. The pressure to maintain a successful image intensifies the pursuit of an idealized body [25,26]. These combined factors contribute to increased dissatisfaction with one’s body image, given that the expectations are frequently high and difficult to achieve [25,26].

The internalization of beauty ideals promoted by social circles and family expectations can lead children and adolescents to constant social comparisons [24,25]. When parents and peers excessively value physical appearance or set high standards for performance in various areas, it generates pressure to meet these expectations [26,57,58,59,60,61]. As these ideals are often unrealistic and difficult to achieve, the result is decreased self-esteem and body image dissatisfaction [26,57,58,59,60,61]. Furthermore, this scoping review verified that preschool-aged females reported greater body dissatisfaction compared to males [60,61]. From an early age, females are often exposed to more intense beauty ideals and aesthetic pressures than males through media and toys that emphasize specific physical appearance standards [60,61]. Additionally, parents and caregivers may reinforce these stereotypes by praising females’ physical appearance more and emphasizing other attributes in males, such as strength [24].

Furthermore, age, gender, economic status, and ethnicity are determining factors in the formation of body image in children and adolescents, as they influence how these individuals perceive and interpret the beauty standards established by society [26,57,58,59,60,61]. With increasing age, there is greater exposure to social influences, which can intensify concerns about physical appearance [24,25]. Gender plays a crucial role, since girls and boys are often socialized differently regarding body expectations [24,25]. Economic status can exacerbate these issues, with individuals from lower classes having less access to resources that promote body satisfaction, such as fashionable clothing or aesthetic care, while ethnicity can mediate the experience of body image through different ideals of beauty and racial discrimination, affecting self-esteem and consequently body image [26,58,59].

### 4.5. Analytical Dimension: Physical Activity and Body Image

The evidence from this dimension indicated that higher levels of physical activity are associated with lower body dissatisfaction in children and adolescents. The explanation for this relationship between physical activity and body image is based on the fact that engaging in physical activity can result in physical changes, including body weight and body composition, leading to an improved body image [66]. This assumption can be supported by arguments from research on the different physical activity choices, as males generally engage in competitive sports and activities that emphasize strength and musculature, while females may be more involved in activities such as dance or gymnastics [66,67].

### 4.6. Analytical Dimension: Personality and Cognitive Thinking for Understanding Body Image

The systematic reviews in this analytical dimension revealed that body dissatisfaction is associated with attentional bias toward stimuli, higher levels of neuroticism, and lower levels of extroversion. The main attentional processes linked to high levels of body dissatisfaction are those related to appearance and eating compared to those with lower levels of concern in these areas [68]. Additionally, individuals with high levels of neuroticism are characterized by greater self-consciousness and vulnerability, making them more sensitive to rejection, which can increase the desire for an ideal body [68]. Furthermore, low levels of extroversion are characterized by greater shyness, less interest in interpersonal interactions, and experiencing negative emotions and sensitivity to social threats [29], making them more susceptible to body dissatisfaction. These results suggest that cognitive thinking and personality (particularly neuroticism and low extroversion) are important correlates of negative body image [29,68]. 

### 4.7. Analytical Dimension: Studies on Body Image with Specific Populations

Regarding studies on body image with specific populations, this scoping review demonstrated that for populations diagnosed with diseases such as cancer, HIV, and lupus, systematic reviews reported greater body dissatisfaction in these subgroups [12,30,31,32,33,34,37]. The diseases investigated in the systematic reviews can affect body image perception in various ways, including physical changes, psychosocial impact, and lifestyle restrictions, contributing to problems such as low self-esteem and eating disorders [37]. These studies work with the age range from 0 to 19 years [12,30,31,32,33,34,36], showing a lack of information from older age groups.

A specific population identified in this scoping review was pregnant and postpartum women (age > 16), where greater body dissatisfaction was reported during this period. Pregnancy and the postpartum period are associated with significant changes in body image perception [36]. Women may face emotional challenges due to physical transformations during pregnancy and social pressure after childbirth to regain their pre-pregnancy shape [35,36,38].

### 4.8. Strengths and Limitations

The strengths of this review include the extensive number of databases and the breadth of information analyzed/reviewed, as well as the systematic analysis of all available information according to each analytical dimension. However, important limitations of this review should be reported, such as the small number of studies aimed at investigating body image assessment methods. Despite the rigorous methodological control adopted in the information search, publication bias cannot be disregarded. Another aspect that should be highlighted concerns the quality of the conduct of the systematic reviews with meta-analyses analyzed.

## 5. Conclusions

It can be concluded that (I) the results highlight that peer influence, physical activity, media, and the school environment play crucial roles in shaping young people’s body image; (II) factors such as sex, age, and socioeconomic context emerge as important variables in understanding body perceptions; and (III) educational interventions and health promotion programs have been shown to be effective in preventing and reducing body dissatisfaction, underscoring the need for multifactorial and collaborative approaches.

## Figures and Tables

**Table 1:** Characteristics of the systematic reviews included, according to the type of review and the dimensions investigated (n = 41).

Dimensions Investigated (n = 7)								
Characteristics of the Studies	Body Image Measurement Instruments (n = 01/2.44%)	Programs and Interventions Focused on Body Image (n = 09/21.95%)	Sociocultural Influences on Body Image (n = 07/17.07%)	Sociodemographic Aspects Related to Body Image (n = 06/14.63%)	Physical Activity and Body Image (n = 06/14.63%)	Personality and Cognitive Thinking to Understand Body Image (n = 02/4.88%)	Studies on Body Image with Specific Populations (n = 10/24.40%)	Total (n = 41/100%)
Type of review	n (%)	n (%)	n (%)	n (%)	n (%)	n (%)	n (%)	
Systematic reviews	00 (00)	04 (44.44)	05 (71.43)	04 (66.67)	05 (83.33)	02 (100)	07 (70.00)	27 (65.85)
Systematic reviews with meta-analyses	01 (100)	05 (55.56)	02 (28.57)	02 (33.33)	01 (16.67)	00 (00)	03(30.00)	14 (34.15)
Year of publication	n (%)	n (%)	n (%)	n (%)	n (%)	n (%)	n (%)	
2006	00 (00)	00 (00)	00 (00)	01 (16.67)	00 (00)	00 (00)	00 (00)	01 (2.43)
2008	00 (00)	00 (00)	01 (14.29)	00 (00)	00 (00)	00 (00)	00 (00)	01 (2.43)
2009	00 (00)	00 (00)	00 (00)	00 (00)	00 (00)	00 (00)	01 (10.00)	01 (2.43)
2011	00 (00)	00 (00)	00 (00)	01 (16.67)	00 (00)	00 (00)	00 (00)	01 (2.43)
2012	00 (00)	00 (00)	00 (00)	00 (00)	00 (00)	00 (00)	01 (10.00)	01 (2.43)
2013	01 (100)	01 (11.11)	00 (00)	00 (00)	00 (00)	00 (00)	01 (10.00)	03 (7.32)
2014	00 (00)	00 (00)	00 (00)	00 (00)	00 (00)	00 (00)	01 (10.00)	01 (2.43)
2015	00 (00)	00 (00)	00 (00)	00 (00)	00 (00)	00 (00)	01 (10.00)	01 (2.43)
2016	00 (00)	00 (00)	00 (00)	02 (33.32)	00 (00)	02 (100)	00 (00)	04 (9.76)
2018	00 (00)	00 (00)	00 (00)	01 (16.67)	03 (50.00)	00 (00)	00 (00)	04 (9.76)
2019	00 (00)	00 (00)	01 (14.28)	00 (00)	00 (00)	00 (00)	00 (00)	01 (2.43)
2020	00 (00)	03 (33.33)	00 (00)	00 (00)	01 (16.66)	00 (00)	02 (20.00)	06 (14.63)
2021	00 (00)	01 (11.11)	02 (28.57)	00 (00)	01 (16.67)	00 (00)	01 (10.00)	06 (14.63)
2022	00 (00)	04 (44.45)	01 (14.29)	00 (00)	01 (16.67)	00 (00)	01 (10.00)	07 (14.70)
2023	00 (00)	00 (00)	02 (28.57)	01 (16.67)	00 (00)	00 (00)	01 (10.00)	04 (9.76)
Geographic location of the corresponding author	n (%)	n (%)	n (%)	n (%)	n (%)	n (%)	n (%)	
North America	00 (00)	00 (00)	02 (28.57)	02 (33.32)	02 (33.33)	01 (50.00)	01 (10.00)	08 (19.51)
South America	01 (100)	00 (00)	01 (14.29)	01 (16.67)	01 (16.67)	00 (00)	02 (20.00)	06 (14.63)
Middle East	00 (00)	01 (11.11)	00 (00)	00 (00)	00 (00)	00 (00)	00 (00)	01 (2.44)
East Asia	00 (00)	00 (00)	00 (00)	00 (00)	00 (00)	00 (00)	01 (10.00)	01 (2.44)
Southeast Asia	00 (00)	02 (22.22)	00 (00)	00 (00)	00 (00)	00 (00)	01 (10.00)	03 (7.32)
Southern Europe	00 (00)	00 (00)	04 (57.14)	01 (00)	02 (33.33)	00 (00)	00 (00)	07 (17.07)
Central Europe	00 (00)	00 (00)	00 (00)	01 (16.67)	01 (16.67)	00 (00)	01 (10.00)	03 (7.32)
Western Europe	00 (00)	03 (33.34)	00 (00)	00 (00)	00 (00)	00 (00)	04 (40.00)	07 (17.07)
Northern Europe	00 (00)	01 (11.11)	00 (00)	00 (00)	00 (00)	00 (00)	00 (00)	01 (2.44)
Australasia	00 (00)	02 (22.22)	00 (00)	01 (16.67)	00 (00)	01 (50.00)	00 (00)	04 (9.76)
Sample	n (%)	n (%)	n (%)	n (%)	n (%)	n (%)	n (%)	
Children	00 (00)	02 (22.22)	00 (00)	02 (33.33)	00 (00)	00 (00)	00 (00)	04 (9.77)
Teenagers	01 (100)	04 (44.45)	06 (85.71)	03 (50.00)	05 (83.33)	02 (100)	05 (50.00)	26 (63.41)
Both	00 (00)	03 (33.33)	01 (14.29)	01 (16.67)	01 (16.67)	00 (00)	05 (50.00)	11 (26.82)
Sample	n (%)	n (%)	n (%)	n (%)	n (%)	n (%)	n (%)	
Women	00 (00)	02 (22.22)	00 (00)	00 (00)	01 (16.67)	00 (00)	02 (20.00)	05 (12.19)
Men	00 (00)	00 (00)	00 (00)	00 (00)	01 (16.67)	00 (00)	00 (00)	01 (2.43)
Both	00 (00)	04 (44.45)	04 (57.14)	05 (83.33)	03 (66.66)	01 (50.00)	02 (20.00)	19 (46.34)
Did not specify	01 (100)	03 (33.33)	03 (42.86)	01 (16.67)	01 (16.67))	01 (50.00)	06 (60.00)	16 (42.04)
Databases searched	n (%)	n (%)	n (%)	n (%)	n (%)	n (%)	n (%)	
01	00 (00)	00 (00)	00 (00)	01 (16.66)	00 (00)	00 (00)	00 (00)	01 (2.43)
02	00 (00)	00 (00)	02 (40.00)	01 (16.66)	02 (33.33)	00 (00)	02 (20.00)	07 (15.11)
03	00 (00)	02 (22.23)	01 (20.00)	01 (16.67)	01 (16.67)	01 (50.00)	00 (00)	06 (14.63)
04	01 (100)	02 (22.22)	00 (00)	01 (16.67)	01 (16.67)	00 (00)	02 (20.00)	07 (15.11)
05	00 (00)	01 (11.11)	03 (40.00)	01 (16.67)	02 (33.33)	00 (00)	02 (20.00)	09 (25.88)
06	00 (00)	01 (11.11)	01 (00)	00 (00)	00 (00)	00 (00)	00 (00)	02 (4.88)
07	00 (00)	02 (22.22)	00 (00)	01 (16.67)	00 (00)	00 (00)	01 (10.00)	04 (9.77)
08	00 (00)	00 (00)	00 (00)	00 (00)	00 (00)	00 (00)	02 (20.00)	02 (4.88)
09	00 (00)	01 (11.11)	00 (00)	00 (00)	00 (00)	01 (50.00)	00 (00)	02 (4.88)
10	00 (00)	00 (00)	00 (00)	00 (00)	00 (00)	00 (00)	01 (10.00)	01 (2.43)
Google Scholar *								
Yes	00 (00)	03 (33.33)	01 (14.29)	01 (16.67)	00 (00)	00 (00)	01 (10.00)	06 (14.63)
No	01 (100)	06 (66.66)	06 (85.71)	05 (83.33)	06 (100)	02 (100)	09 (90.00)	35 (85.37)
Total original articles mapped in reviews	n (%)	n (%)	n (%)	n (%)	n (%)	n (%)	n (%)	
	28	193	246	316	137	65	523	1508

Note. * Google Scholar was considered gray literature even when the review itself classified it as a database.

## Supplementary Materials

The following supporting information can be downloaded at: https://www.mdpi.com/article/10.3390/ejihpe14100179/s1, Table S1: Description of systematic reviews and meta-analyses on body image in children and adolescents (zero to 19 years of age); Table S2: Description of objectives, study locations, assessment instruments and main results of systematic reviews on body image in children and adolescents (zero to 19 years of age); Table S3: Assessment of the risk of bias of the articles included in the review.
