# Supplementary material for: Comprehensive Scoping Review on Body Image Perceptions and Influences in Children and Adolescents
Source: Eur J Investig Health Psychol Educ. 2024 Oct 4;14(10):2716–34. doi: 10.3390/ejihpe14100179 (PMC11507315; doi:10.3390/ejihpe14100179)
Supplement: Supplementary file 1 [file ejihpe-14-00179-s001.zip › ejihpe-3086629-supplementary.pdf]

SUPPLEMENTARY DOCUMENT

List of descriptors:

Block 1: *"body image" OR "self perception" OR "self image" OR "body satisfaction" OR "body dissatisfaction" OR "self esteem" OR "body perception" OR "weight perception"*

Block 2: *child* OR adolesc* OR student OR youth OR adolescent* OR adolescence OR teen OR teenage OR teenager OR scholar OR "young people" OR "school children" OR "school teenager" OR young*

Block 3: *"Meta-Analysis" OR "Systematic Review"*

1 - PubMed via National Library of Medicine (MEDLINE). Search carried out on: 10/20/2023		
**Block**	**Descriptors**	**Articles**
1	*"body image" OR "self perception" OR "self image" OR "body satisfaction" OR "body dissatisfaction" OR "self esteem" OR "body perception" OR "weight perception"*	
2	*child* OR adolesc* OR student OR youth OR adolescent* OR adolescence OR teen OR teenage OR teenager OR scholar OR "young people" OR "school children" OR "school teenager" OR young*	
3	*"Meta-Analysis" OR "Systematic Review"*	
"body image" OR "self perception" OR "self image" OR "body satisfaction" OR "body dissatisfaction" OR "self esteem" OR "body perception" OR "weight perception" AND child OR adolesc* OR student OR youth OR adolescent* OR adolescence OR teen OR teenage OR teenager OR scholar OR "young people" OR "school children" OR "school teenager" OR young AND "Meta-Analysis" OR "Systematic Review"*		760

2 – Scopus. Search carried out on: 10/20/2023.		
**Block**	**Descriptors**	**Articles**

1	*"body image" OR "self perception" OR "self image" OR "body satisfaction" OR "body dissatisfaction" OR "self esteem" OR "body perception" OR "weight perception"*	
2	*child* OR adolesc* OR student OR youth OR adolescent* OR adolescence OR teen OR teenage OR teenager OR scholar OR "young people" OR "school children" OR "school teenager" OR young*	
3	*"Meta-Analysis" OR "Systematic Review"*	
(TITLE-ABS-KEY ("body image" OR "self perception" OR "self image" OR "body satisfaction" OR "body dissatisfaction" OR "self esteem" OR "body perception" OR "weight perception") AND TITLE-ABS-KEY (: AND child* OR adolesc* OR student OR youth OR adolescent* OR adolescence OR teen OR teenage OR teenager OR scholar OR "young people" OR "school children" OR "school teenager" OR young) AND TITLE-ABS-KEY ("Meta-Analysis" OR "Systematic Review")) AND (EXCLUDE (SUBJAREA , "AGRI") OR EXCLUDE (SUBJAREA , "COMP") OR EXCLUDE (SUBJAREA , "ENGI") OR EXCLUDE (SUBJAREA , "BUSI") OR EXCLUDE (SUBJAREA , "ECON") OR EXCLUDE (SUBJAREA , "VETE") OR EXCLUDE (SUBJAREA , "MATH") OR EXCLUDE (SUBJAREA , "MATE") OR EXCLUDE (SUBJAREA , "CHEM") OR EXCLUDE (SUBJAREA , "IMMU") OR EXCLUDE (SUBJAREA , "PHAR") OR EXCLUDE (SUBJAREA , "DENT") OR EXCLUDE (SUBJAREA , "ENVI") OR EXCLUDE (SUBJAREA , "BIOC") OR EXCLUDE (SUBJAREA , "NEUR")) AND (LIMIT-TO (DOCTYPE , "re") OR LIMIT-TO (DOCTYPE , "ar")) AND (LIMIT-TO (EXACTKEYWORD , "Human") OR LIMIT-TO (EXACTKEYWORD , "Humans") OR LIMIT-TO (EXACTKEYWORD , "Systematic Review") OR LIMIT-TO (EXACTKEYWORD , "Adolescent") OR LIMIT-TO (EXACTKEYWORD , "Female") OR LIMIT-TO (EXACTKEYWORD , "Self Esteem") OR LIMIT-TO (EXACTKEYWORD , "Child") OR LIMIT-TO (EXACTKEYWORD , "Male") OR LIMIT-TO (EXACTKEYWORD , "Adult") OR LIMIT-TO (EXACTKEYWORD , "Psychology") OR LIMIT-TO (EXACTKEYWORD , "Meta Analysis") OR LIMIT-TO (EXACTKEYWORD , "Depression") OR LIMIT-TO (EXACTKEYWORD , "Self Concept") OR LIMIT-TO (EXACTKEYWORD , "Body Image") OR LIMIT-TO (EXACTKEYWORD , "Mental Health") OR LIMIT-TO (EXACTKEYWORD , "Anxiety") OR LIMIT-TO (EXACTKEYWORD , "Meta-analysis") OR LIMIT-TO (EXACTKEYWORD , "Physical Activity") OR LIMIT-TO (EXACTKEYWORD , "Self-esteem") OR LIMIT-TO (EXACTKEYWORD , "Adolescents") OR LIMIT-TO (EXACTKEYWORD , "Body Mass") OR LIMIT-TO (EXACTKEYWORD , "Children") OR LIMIT-TO (EXACTKEYWORD , "Preschool Child") OR LIMIT-TO (EXACTKEYWORD , "Child, Preschool") OR LIMIT-TO (EXACTKEYWORD , "Adolescence") OR LIMIT-TO (EXACTKEYWORD , "Body Weight") OR LIMIT-TO (EXACTKEYWORD , "Body Dissatisfaction") OR LIMIT-TO (		1088

EXACTKEYWORD , "Adolescent Behavior") OR LIMIT-TO (EXACTKEYWORD , "Age") OR LIMIT-TO (EXACTKEYWORD , "Feeding And Eating Disorders") OR LIMIT-TO (EXACTKEYWORD , "Meta Analysis (topic)") OR LIMIT-TO (EXACTKEYWORD , "Attitude To Health"))	

3 - Scientific Electronic Library Online (SciELO). Search carried out on: 10/20/2023.		
**Block**	**Descriptors**	**Articles**
1	*“body image” OR “self perception” OR “self image” OR “body satisfaction” OR “body dissatisfaction” OR “self esteem” OR “body perception” OR “weight perception”*	
2	*child* OR adolesc* OR student OR youth OR adolescent* OR adolescence OR teen OR teenage OR teenager OR scholar OR “young people” OR “school children” OR “school teenager” OR young*	
3	*“Meta-Analysis” OR “Systematic Review”*	
(“body image” OR “self perception” OR “self image” OR “body satisfaction” OR “body dissatisfaction” OR “self esteem” OR “body perception” OR “weight perception”) AND (child* OR adolesc* OR student OR youth OR adolescent* OR adolescence OR teen OR teenage OR teenager OR scholar OR “young people” OR “school children” OR “school teenager” OR young) AND (“Meta-Analysis” OR “Systematic Review”)		00

4 - PsycINFO via American Psychological Association (APA). Search carried out on: 10/20/2023.		
**Block**	**Descriptors**	**Articles**
1	*“body image” OR “self perception” OR “self image” OR “body satisfaction” OR “body dissatisfaction” OR “self esteem” OR “body perception” OR “weight perception”*	
2	*child* OR adolesc* OR student OR youth OR adolescent* OR adolescence OR teen OR teenage OR teenager OR scholar OR “young people” OR “school children” OR “school teenager” OR young*	
3	*“Meta-Analysis” OR “Systematic Review”*	
Results for **Any Field**: “body image” OR “self perception” OR “self image” OR “body satisfaction” OR “body dissatisfaction” OR “self esteem” OR “body perception” OR “weight perception” *AND* **Any Field**: child* OR adolesc* OR student OR youth OR adolescent* OR adolescence OR teen OR teenage OR teenager OR scholar OR “young		227

people" OR "school children" OR "school teenager" OR young *AND* **Any Field**: "Meta-Analysis" OR "Systematic Review" *AND* **Methodology**: Meta Analysis	

5 - *Cochrane Library. Search carried out on: 10/20/2023.*		
**Block**	**Descriptors**	**Articles**
1	*"body image" OR "self perception" OR "self image" OR "body satisfaction" OR "body dissatisfaction" OR "self esteem" OR "body perception" OR "weight perception"*	
2	*child* OR adolesc* OR student OR youth OR adolescent* OR adolescence OR teen OR teenage OR teenager OR scholar OR "young people" OR "school children" OR "school teenager" OR young*	
3	*"Meta-Analysis" OR "Systematic Review"*	
matching "body image" OR "self perception" OR "self image" OR "body satisfaction" OR "body dissatisfaction" OR "self esteem" OR "body perception" OR "weight perception" in Title Abstract Keyword AND child* OR adolesc* OR student OR youth OR adolescent* OR adolescence OR teen OR teenage OR teenager OR scholar OR "young people" OR "school children" OR "school teenager" OR young in Title Abstract Keyword AND "Meta-Analysis" OR "Systematic Review" in Title Abstract Keyword - (Word variations have been searched)		10

6 - LILACS via Virtual Health Library. Search carried out on: 10/20/2023.		
**Block**	**Descriptors**	**Articles**
1	*"body image" OR "self perception" OR "self image" OR "body satisfaction" OR "body dissatisfaction" OR "self esteem" OR "body perception" OR "weight perception"*	
2	*child* OR adolesc* OR student OR youth OR adolescent* OR adolescence OR teen OR teenage OR teenager OR scholar OR "young people" OR "school children" OR "school teenager" OR young*	
3	*"Meta-Analysis" OR "Systematic Review"*	
"body image" OR "self perception" OR "self image" OR "body satisfaction" OR "body dissatisfaction" OR "self esteem" OR "body perception" OR "weight perception" [words] and child* OR adolesc* OR student OR youth OR adolescent* OR adolescence OR teen OR teenage OR teenager OR scholar OR "young people" OR "school children" OR		00

“school teenager” OR young [words] and “Meta-Analysis” OR “Systematic Review” [words]	

7 - Cumulative Index to Nursing and Allied Health Literature (CINAHL), via EBSCOhost. Search carried out on: 10/20/2023.		
**Block**	**Descriptors**	**Articles**
1	*“body image” OR “self perception” OR “self image” OR “body satisfaction” OR “body dissatisfaction” OR “self esteem” OR “body perception” OR “weight perception”*	
2	*child* OR adolesc* OR student OR youth OR adolescent* OR adolescence OR teen OR teenage OR teenager OR scholar OR “young people” OR “school children” OR “school teenager” OR young*	
3	*“Meta-Analysis” OR “Systematic Review”*	
(“body image” OR “self perception” OR “self image” OR “body satisfaction” OR “body dissatisfaction” OR “self esteem” OR “body perception” OR “weight perception”) AND (child* OR adolesc* OR student OR youth OR adolescent* OR adolescence OR teen OR teenage OR teenager OR scholar OR “young people” OR “school children” OR “school teenager” OR young) AND (“Meta-Analysis” OR “Systematic Review”)		191

8 - SPORTDiscus via EBSCOhost. Search carried out on: 10/20/2023.		
**Block**	**Descriptors**	**Articles**
1	*“body image” OR “self perception” OR “self image” OR “body satisfaction” OR “body dissatisfaction” OR “self esteem” OR “body perception” OR “weight perception”*	
2	*child* OR adolesc* OR student OR youth OR adolescent* OR adolescence OR teen OR teenage OR teenager OR scholar OR “young people” OR “school children” OR “school teenager” OR young*	
3	*“Meta-Analysis” OR “Systematic Review”*	
(“body image” OR “self perception” OR “self image” OR “body satisfaction” OR “body dissatisfaction” OR “self esteem” OR “body perception” OR “weight perception”) AND (child* OR adolesc* OR student OR youth OR adolescent* OR adolescence OR teen OR teenage OR teenager OR scholar OR “young people” OR “school children” OR “school teenager” OR young) AND (“Meta-Analysis” OR “Systematic Review”)		35

9 - Web of Science. Search carried out on: 10/20/2023.		
**Block**	**Descriptors**	**Articles**
1	*"body image" OR "self perception" OR "self image" OR "body satisfaction" OR "body dissatisfaction" OR "self esteem" OR "body perception" OR "weight perception"*	
2	*child* OR adolesc* OR student OR youth OR adolescent* OR adolescence OR teen OR teenage OR teenager OR scholar OR "young people" OR "school children" OR "school teenager" OR young*	
3	*"Meta-Analysis" OR "Systematic Review"*	
(body image" OR "self perception" OR "self image" OR "body satisfaction" OR "body dissatisfaction" OR "self esteem" OR "body perception" OR "weight perception") AND (child* OR adolesc* OR student OR youth OR adolescent* OR adolescence OR teen OR teenage OR teenager OR scholar OR "young people" OR "school children" OR "school teenager" OR young) AND ("Meta-Analysis" OR "Systematic Review")		946

**Supplementary Table S1**. Description of systematic reviews and meta-analyses on body image in children and adolescents (zero to 19 years of age)						
TITLE/YEAR OF PUBLICATION	AUTHORS	TYPE OF SELECTED STUDIES	NUMBER OF STUDIES INCLUDED	TOTAL NUMBER OF SUBJECTS	DATABASES SEARCHED	METANALYSIS
**Body Image Measurement Instruments**						
O uso de escalas de silhuetas na avaliação da satisfação corporal de adolescentes: revisão sistemática da literatura /2013	Marcela Guimarães Côrtes et al.	cross-sectional, before-after (pre-post) studies with no control group and validation study	28	37.229<br>not specified: 37.229	04 (LILACS, PubMed, MEDLINE and SciELO)	Yes, a meta-analysis was carried out
**Programs and Interventions focused on Body Image**						
A systematic review of interventions aiming to promote positive body image in children and adolescents/ 2022	Ella Guest et al.	randomized clinical trial, randomized trial and observational cohort	13	4.645<br>female: 1.040<br>male: 237<br>not specified: 3.371	06 (CINAHL, Medline, PsychINFO, Wiley Online Library, SCOPUS and grey literature)	No meta-analysis was performed
Effects of Media Health Literacy SchoolBased Interventions on Adolescents' Body Image Concerns, Eating	Areeg A. Zuair et al.	randomized clinical trial	16	4.760<br>female: 2.979<br>male: 1.218	03 (PubMed, ProQuest, and PsycINFO)	Yes, a meta-analysis was carried out

**Supplementary Table S1**. Description of systematic reviews and meta-analyses on body image in children and adolescents (zero to 19 years of age)						
TITLE/YEAR OF PUBLICATION	AUTHORS	TYPE OF SELECTED STUDIES	NUMBER OF STUDIES INCLUDED	TOTAL NUMBER OF SUBJECTS	DATABASES SEARCHED	METANALYSIS
Concerns, and Thin Internalization Attitudes: A Systematic Review and Meta-Analysis / 2020						
A systematic review exploring body image programmes and interventions in physical education/ 2022	Charlotte Kerner et al.	before-after (pre-post) studies with no control group and randomized clinical trial	19	5.852<br>female: 1.680<br>male: 983<br>not specified: 3.189	07 (Web of Science, SCOPUS, EBSCO, PsycINFO, MEDLINE, Science Direct, and SportDiscus)	No meta-analysis was performed
Pediatric obesity treatment, self-esteem, and body image: A systematic review with meta-analysis/2020	Megan L. Gow et al.	randomized clinical trial	64	10.471<br>sex was not specified	04 (MEDLINE, EMBASE, Cochrane Library, and PsychINFO)	Yes, a meta-analysis was carried out
Research Review: Effectiveness of universal eating disorder prevention interventions in improving body image among	Joelle Yan Xin Chua et al.	randomized clinical trial	24	9.093<br>sex was not specified	09 (PubMed, Cochrane Library, Embase, American Psychological Association, Scopus,	Yes, a meta-analysis was carried out

**Supplementary Table S1**. Description of systematic reviews and meta-analyses on body image in children and adolescents (zero to 19 years of age)						
TITLE/YEAR OF PUBLICATION	AUTHORS	TYPE OF SELECTED STUDIES	NUMBER OF STUDIES INCLUDED	TOTAL NUMBER OF SUBJECTS	DATABASES SEARCHED	METANALYSIS
children: a systematic review and meta-analysis/2020					Cumulative Index to Nursing and Allied Health Literature, Web of Science, ProQuest Dissertations and Theses, and Mednar)	
Systematic Review of Digital Interventions for Adolescent and Young Adult Women's Body Image/2022	Ciara Mahon et al.	randomized clinical trial	15	5.011<br>female: 5.011	03 (PsychINFO, Web of Science and Pubmed)	No meta-analysis was performed
School-Based Interventions Improve Body Image and Media Literacy in Youth: A Systematic Review and Meta-Analysis/ 2022	Martina Kurz et al.	randomised controlled trial, Cluster	17	8897 participants with data for post-assessment, and 7392 participants with data for post-assessment and follow-up.<br>women: -	04 (PsycINFO, Medline, Web of Science Core Collection e Cochrane Central Register of Controlled Trials)	Yes, a meta-analysis was carried out

**Supplementary Table S1**. Description of systematic reviews and meta-analyses on body image in children and adolescents (zero to 19 years of age)						
TITLE/YEAR OF PUBLICATION	AUTHORS	TYPE OF SELECTED STUDIES	NUMBER OF STUDIES INCLUDED	TOTAL NUMBER OF SUBJECTS	DATABASES SEARCHED	METANALYSIS
				male: -<br>not specified: 8897 participants with data for post-assessment, and 7392 participants with data for post-assessment and follow-up		
The effect of cognitive behavior therapy on body image and self-esteem in female adolescents: a systematic review and meta-analysis/2021	Nasrin Zamiri-Miandoab et al.	Randomized controlled trials, quasi-experimental studies	09	429<br>women: -<br>male: -<br>not specified: 429	07 (Cochrane, web of science, EBSCO, PubMed, Google Scholar and Persian databases SID, MagIran)	Yes, a meta-analysis was carried out
What works in secondary schools? A systematic review of classroom-based	Zali Yager et al.	Intervention studies	16	4352<br>women: 2819	05 (EBSCOHOST, Medline, PsycINFO,	No meta-analysis was performed

**Supplementary Table S1**. Description of systematic reviews and meta-analyses on body image in children and adolescents (zero to 19 years of age)						
TITLE/YEAR OF PUBLICATION	AUTHORS	TYPE OF SELECTED STUDIES	NUMBER OF STUDIES INCLUDED	TOTAL NUMBER OF SUBJECTS	DATABASES SEARCHED	METANALYSIS
body image programs/2013				male: 959<br>not specified: 574	Current Contents, Google Scholar)	
**Sociocultural influences on body image**						
A systematic review on the effects of media disclaimers on young women's body image and mood/2019	Sarah E. McComb et al.	randomized clinical trial	15	765<br>female: 635<br>male: 130	02 (PsycINFO and Medline/ PubMed)	No meta-analysis was performed
Effects of fitspiration content on body image: a systematic review/2022	Flávio Jerónimo et al.	cross-sectional and randomized clinical trial	20	2.352<br>female: 2.108<br>male: 244	03 (Pubmed, PsycINFO and Google Scholar)	No meta-analysis was performed
Internalization of body shape ideals and body dissatisfaction: A systematic review and meta-analysis/2021	Adrian Paterna et al.	cross-sectional and before-after (pre-post) studies with no control group	78	16.300<br>female: 14.061<br>male: 1.541<br>not specified: 698	05 (MEDLINE, PsycINFO, Web of Science, SciELO, and Dissertations & Theses Global)	Yes, a meta-analysis was carried out

Supplementary Table S1. Description of systematic reviews and meta-analyses on body image in children and adolescents (zero to 19 years of age)

TITLE/YEAR OF PUBLICATION	AUTHORS	TYPE OF SELECTED STUDIES	NUMBER OF STUDIES INCLUDED	TOTAL NUMBER OF SUBJECTS	DATABASES SEARCHED	METANALYSIS
Uso de redes sociales y factores de riesgo para el desarrollo de trastornos relacionados con la alimentación en Espana: una revisión sistemática/2023	Stephany Bajana Marí et al.	Cross-sectional	12	No information was reported on the specific number of participants aged 0 to 19 years and sex.	03 (PubMed, Scopus and Web of Science)	No meta-analysis was performed
The Role of the Media in Body Image Concerns Among Women: A Meta-Analysis of Experimental and Correlational Studies/2008	Shelly Grabe et al.	No reported	77	No information was reported on the specific number of participants aged 0 to 19 years and sex.	02 (PsycINFO and Web of Science)	Yes, a meta-analysis was carried out
Prevalence of body weight dissatisfaction among adolescents: a systematic review/2023	Mariana Contiero San Martini et al.	Cross-sectional	34	96.140<br>women: -<br>male: -<br>not specified: 96140	05 (PubMed, Ovid, The Cumulative Index to Nursing and Allied Health Literature e American	No meta-analysis was performed

**Supplementary Table S1**. Description of systematic reviews and meta-analyses on body image in children and adolescents (zero to 19 years of age)						
TITLE/YEAR OF PUBLICATION	AUTHORS	TYPE OF SELECTED STUDIES	NUMBER OF STUDIES INCLUDED	TOTAL NUMBER OF SUBJECTS	DATABASES SEARCHED	METANALYSIS
					Psychological Association)	
The Meaning and Factors That Influence the Concept of Body Image: Systematic Review and Meta-Ethnography from the Perspectives of Adolescents/2021	Glòria Tort-Nasarre et al.	Qualitative studies	10	518<br>women: 376<br>male: 142<br>not specified: -	06 (Web of Science, PubMed, PsycINFO, CINAHL, Scopus, and the Cochrane Register)	No meta-analysis was performed
**Sociodemographic aspects related to Body Image**						
Normative body image development: A longitudinal meta-analysis of mean-level change /2023	Emilie Lacroix et al.	before-after (pre-post) studies with no control group	143	128.254<br>women: 67670<br>male: 44691<br>not specified: 15633	05 (EMBASE, Medline, Web of Science, PsycINFO and ProQuest Dissertation & Theses)	Yes, a meta-analysis was carried out
Percepção da imagem corporal e nível socioeconômico em	Érico Felden Pereira et al.	No reported	11	12.126<br>women: 4080	02 (PubMed and SciELO)	No meta-analysis was performed

**Supplementary Table S1**. Description of systematic reviews and meta-analyses on body image in children and adolescents (zero to 19 years of age)						
TITLE/YEAR OF PUBLICATION	AUTHORS	TYPE OF SELECTED STUDIES	NUMBER OF STUDIES INCLUDED	TOTAL NUMBER OF SUBJECTS	DATABASES SEARCHED	METANALYSIS
adolescentes: revisão sistemática/2011				male: -<br>not specified: 8046		
Body image perception of African immigrants in Europe/2016	Stefania Toselli et al.	cross-sectional	26	3.678<br>female: 1.893<br>male: 1.554<br>not specified: 231	03 (PubMed, PsycINFO and Google Scholar)	No meta-analysis was performed
Ethnicity and Body Dissatisfaction Among Women in the United States: A Meta-Analysis/2006	Shelly Grabe, et al.	cross-sectional, before-after (pre-post) studies with no control group, observational cohort and randomized clinical trial	98	17.653<br>sex was not specified	01 (PsycINFO)	Yes, a meta-analysis was carried out
Perception of body size and dissatisfaction in children aged 3 to 6: a systematic review/2018	María-Pilar León et al.	Quantitative	22	5548<br>women: 2097<br>male: 3214<br>not specified: 237	07 (Medline, SportDiscus, Scopus, ScienceDirect, Dialnet, ProQuest and EBSCO)	No meta-analysis was performed

**Supplementary Table S1**. Description of systematic reviews and meta-analyses on body image in children and adolescents (zero to 19 years of age)						
TITLE/YEAR OF PUBLICATION	AUTHORS	TYPE OF SELECTED STUDIES	NUMBER OF STUDIES INCLUDED	TOTAL NUMBER OF SUBJECTS	DATABASES SEARCHED	METANALYSIS
A systematic review of body dissatisfaction and sociocultural messages related to the body among preschool children / 2016	Gemma Tatangelo et al.	cross-sectional, controlled intervention and before-after (pre-post) studies with no control group	16	3.655<br>female: 1.842<br>male: 1.671<br>not specified:142	04 (Medline Complete, SCOPUS, Web of Science, and PsychINFO)	No meta-analysis was performed
**Physical Activity and Body Image**						
Physical activity and body image among men and boys: A meta-analysis/2018	Rebecca Bassett-Gunter et al.	cross-sectional, randomized clinical trial, before-after (pre-post) studies with no control group	35	No information was reported on the specific number of participants aged 0 to 19 years and sex.	05 (PsycINFO , Medline, SportDiscus , Education Resources Information Center (ERIC) and ProQuest Dissertations and Theses)	Yes, a meta-analysis was carried out

Supplementary Table S1. Description of systematic reviews and meta-analyses on body image in children and adolescents (zero to 19 years of age)

TITLE/YEAR OF PUBLICATION	AUTHORS	TYPE OF SELECTED STUDIES	NUMBER OF STUDIES INCLUDED	TOTAL NUMBER OF SUBJECTS	DATABASES SEARCHED	METANALYSIS
Effects of exercise motivations on body image and eating habits/behaviours: A systematic review/ 2020	Inês Panão et al.	cross-sectional study and before-after (pre-post) studies with no control group, randomized clinical trial	26	1.892<br>female: 918<br>male: 454<br>not specified: 520	03 (PubMed, PsycINFO and SportDiscus)	No meta-analysis was performed
Body image of healthy adolescent women and its association with physical activity: a systematic review/2018	Roberta Luksevicius Rica et al.	cross-sectional and randomized clinical trial	18	6.358<br>female: 6.358	05 (Pubmed, MedLine, Science Direct, Sport Discus and Scielo)	No meta-analysis was performed
Body Image and its Role in Physical Activity: A Systematic Review / 2021	Cassidy M. Foley Davelaar	cross-sectional, before-after (pre-post) studies with no control group, observational cohort, systematic review, exploratory study, randomized clinical trial and review article	22	7.767<br>gender was not specified	02 (PubMed and MEDLINE)	No meta-analysis was performed

**Supplementary Table S1**. Description of systematic reviews and meta-analyses on body image in children and adolescents (zero to 19 years of age)						
TITLE/YEAR OF PUBLICATION	AUTHORS	TYPE OF SELECTED STUDIES	NUMBER OF STUDIES INCLUDED	TOTAL NUMBER OF SUBJECTS	DATABASES SEARCHED	METANALYSIS
Physical Activity and Body Image Perception in Adolescents: A Systematic Review/2022	Emanuela Gualdi-Russo et al.	Cross-sectional and before-after (pre-post) studies with no control group	28	44.489<br>women: 23608<br>male: 20109<br>not specified: 772	02 (PubMed and Web of Science)	No meta-analysis was performed
The Influence of Dance Practice on the Body Image of Adolescents: A Systematic Review/2018	Lilian Alves Costa Monteiro et al.	Cross-sectional, Experimental	08	769<br>women: 565<br>male: 204<br>not specified: -	04 (PubMed, Web of Science, Scopus and Virtual Health Library)	No meta-analysis was performed
**Personality and Cognitive Thinking to understand Body Image**						
Personality and body image: A systematic review /2016	Mark S. Allen et al.	cross-sectional	33	Studies with 18 to 45 years of age were included. No information was reported on the specific number of participants aged 0 to 19 years and sex.	09 (PubMed, Web of Science, psycINFO, psycARTICLES, SPORTDiscus, MEDLINE, ERIC via EBSCO, ProQuest, and Scopus)	No meta-analysis was performed

**Supplementary Table S1**. Description of systematic reviews and meta-analyses on body image in children and adolescents (zero to 19 years of age)						
TITLE/YEAR OF PUBLICATION	AUTHORS	TYPE OF SELECTED STUDIES	NUMBER OF STUDIES INCLUDED	TOTAL NUMBER OF SUBJECTS	DATABASES SEARCHED	METANALYSIS
Cognitive biases to appearance-related stimuli in body dissatisfaction: A systematic review/2016	Rachel F. Rodgers et al.	randomized clinical trial	32	2.463<br>female: 1.941<br>male: 109<br>not specified: 413	03 (PsycInfo, PubMed and ScienceDirect)	No meta-analysis was performed
**Studies on Body Image with Specific Populations**						
Body image of children and adolescents with cancer: A systematic review/ 2009	Sheng-Yu Fan et al.	cross-sectional and before-after (pre-post) studies with no control group	32	3.778<br>sex was not specified	05 (BNI, CINAHL, MEDLINE, PsychInfo, and PubMed)	No meta-analysis was performed
Body image of children and adolescents with chronic illness: A meta-analytic comparison with healthy peers/2013	M. Pinquart	cross-sectional	330	30.209<br>sex was not specified	04 (Google Scholar, MEDLINE, PSNYDEX, PSYCINFO)	Yes, a meta-analysis was carried out
Body image of children and adolescents with cancer: A metasynthesis	Mei-Yin Lee et al.	case study, longitudinal qualitative research, longitudinal descriptive	8	117<br>sex was not specified	07 (PubMed, CINAHL, Scirus, Mednar, ProQuest Dissertations and	No meta-analysis was performed

**Supplementary Table S1**. Description of systematic reviews and meta-analyses on body image in children and adolescents (zero to 19 years of age)						
TITLE/YEAR OF PUBLICATION	AUTHORS	TYPE OF SELECTED STUDIES	NUMBER OF STUDIES INCLUDED	TOTAL NUMBER OF SUBJECTS	DATABASES SEARCHED	METANALYSIS
on qualitative research findings/ 2012		qualitative, phenomenological research, qualitative inquiry, qualitative study			theses, Chinese electronic theses and dissertation systems, and Chinese electronic periodical services)	
Body Image Problems in Individuals with Type 1 Diabetes: A Review of the Literature/2022	Alda Troncone et al.	cross-sectional, case-control and before-after (pre-post) studies with no control group	51	19.455<br>sex was not specified	05 (PsycInfo, PsycArticles, PsycCRITIQUES, Pubmed, and Scopus)	No meta-analysis was performed
Body image in children and adolescents diagnosed with the human immunodeficiency virus: a systematic review / 2023	Suellem Zanlorenci et al.	cross-sectional study	4	473<br>female: 89<br>male: 71<br>not specified: 313	08 (PubMed/MEDLINE, Web of Science, Scopus, SPORTDiscus, LILACS, SciELO, PsycINFO and CINAHL)	No meta-analysis was performed

**Supplementary Table S1**. Description of systematic reviews and meta-analyses on body image in children and adolescents (zero to 19 years of age)						
TITLE/YEAR OF PUBLICATION	AUTHORS	TYPE OF SELECTED STUDIES	NUMBER OF STUDIES INCLUDED	TOTAL NUMBER OF SUBJECTS	DATABASES SEARCHED	METANALYSIS
Association between overweight/obesity with depression, anxiety, low self-esteem, and body dissatisfaction in children and adolescents: a systematic review and meta-analysis of observational studies/2020	Maedeh Moradi et al.	cross-sectional, observational cohort	28	179.306<br>female: 64.485<br>male: 14.048<br>not specified: 100.773	02 (PubMed and Scopus)	Yes, a meta-analysis was carrie out
Body Image in Adolescent Pregnancy/ 2015	Alina Zaltzman et al.	case-control, retrospective exploratory and before-after (pre-post) studies with no control group	6	349<br>female: 349	02 (MEDLINE and PsycINFO)	No meta-analysis was performed
A systematic review examining the association between female body image and the intention, initiation and duration of postpartum infant feeding	Alexandra Grace Morley-Hewitt et al.	Cross-sectional and before-after (pre-post) studies with no control group	09	No information was reported on the specific number of participants aged 0 to 19 years and sex.	10 (ZETOC, Web of Science, PsychAR-TICLES, Scopus, Cochrane Library, Medline, ScienceDirect,	No meta-analysis was performed

**Supplementary Table S1**. Description of systematic reviews and meta-analyses on body image in children and adolescents (zero to 19 years of age)						
TITLE/YEAR OF PUBLICATION	AUTHORS	TYPE OF SELECTED STUDIES	NUMBER OF STUDIES INCLUDED	TOTAL NUMBER OF SUBJECTS	DATABASES SEARCHED	METANALYSIS
methods (breastfeeding vs bottle-feeding)/2020					CINAHL e ProQuest Nursing Journals)	
Self-concept and body image of people living with lupus: A systematic review/2021	Larissa Rodrigues et al.	Cross-sectional, quantitative approach	22	No information was reported on the specific number of participants aged 0 to 19 years and sex.	08 (PubMed, CINAHL, Excerpta Medica, SCOPUS, Web of Science, Medline, Medline Complete e Academic Search Premier)	No meta-analysis was performed
Women's experiences of their pregnancy and postpartum body image: a systematic review and meta-synthesis/2014	Emma L. Hodgkinson et al.	Qualitative studies	17	No information was reported on the specific number of participants aged 0 to 19 years.	04 (PsycINFO, MEDLINE, Web of Knowledge and Embase)	Yes, a meta-analysis was carried out

**Supplementary Table S2.** Description of objectives, study locations, assessment instruments and main results of systematic reviews on body image in children and adolescents (zero to 19 years of age)			
TITLE	OBJECTIVE	BODY IMAGE ASSESSMENT INSTRUMENTS	KEY RESULT
**Body Image Measurement Instruments**			
O uso de escalas de silhuetas na avaliação da satisfação corporal de adolescentes: revisão sistemática da literatura	Synthesize studies on adolescent body satisfaction, focusing on the use of the silhouette scale	Silhouette Scale Stunkard et al., Tiggemann and Pennington's Silhouette scale.	The majority compared satisfaction versus dissatisfaction, using the chi-square test, and did not consider possible confounding variables. Among the 18 studies included in the meta-analysis, the prevalence of dissatisfaction varied from 32,2% a 83%, being observed not only great heterogeneity between them, even stratifying into subgroups, as well as the lack of relevant methodological information.
**Programs and Interventions focused on Body Image**			
A systematic review of interventions aiming to promote positive body image in children and adolescents	To determine the evidence of efficacy of interventions aiming to promote positive body image in children and young people aged 18 years and below.	Body Appreciation Scale - Portuguese version; Body-Esteem Scale for Adolescents and Adults; Body Appreciation Scale; The Appearance Esteem and Weight Esteem subscales of the Body Esteem Scale for Adults and Adolescents; Body appreciation X'X'Scale 2-Children; The Body Appreciation Scale-2; The Appearance Esteem subscale of the Body Esteem Scale for Children and	The studies evaluated body appreciation, body-esteem, and embodiment. Studies using cognitive dissonance, peer support, and psychoeducation had evidence of improving body appreciation and body-esteem in adolescent girls. However, evidence of efficacy for younger children and boys was lacking and the studies ranged in methodological quality. Further research should rigorously evaluate positive

**Supplementary Table S2.** Description of objectives, study locations, assessment instruments and main results of systematic reviews on body image in children and adolescents (zero to 19 years of age)			
TITLE	OBJECTIVE	BODY IMAGE ASSESSMENT INSTRUMENTS	KEY RESULT
		The Body Appreciation Scale-2 for Children; The Body Appreciation Scale-2; Experience of Embodiment Scale; Functionality Satisfaction subscale of Embodied Image Scale.	body image interventions using second-generation measures that assess specific components of positive body image and consider how to promote positive body image in young children and boys.
Effects of Media Health Literacy School Based Interventions on Adolescents' Body Image Concerns, Eating Concerns, and Thin Internalization Attitudes: A Systematic Review and Meta-Analysis	To investigate the effects of media health literacy (MHL) interventions in school settings on adolescents' body image.	No reported	The meta-analysis of effect sizes for three outcomes of interest related to body image showed consistent changes in the desired negative direction as expected: body image concerns eating concerns thin-internalization attitudes; and combined Homogeneity analysis for the combined effect size and moderator analyses for gender composition of intervention group, number of overall intervention sessions, number of MHL specific intervention sessions within the overall intervention, and time length of MHL intervention session all showed absence of moderators. However, all study samples except one were of European origin and so caution should be exercised when generalizing to other cultures that may have different standards of female ideal body type.

Supplementary Table S2. Description of objectives, study locations, assessment instruments and main results of systematic reviews on body image in children and adolescents (zero to 19 years of age)

TITLE	OBJECTIVE	BODY IMAGE ASSESSMENT INSTRUMENTS	KEY RESULT
A systematic review exploring body image programmes and interventions in physical education	To systematically review the evidence on the content and effectiveness of physical education-based body image or body-focused programmes published between 2000 and 2021	Self-Description questionnaire, Body Attitudes Questionnaire, Body Dissatisfaction Scale in Adolescents, Children's Physical Self-Perceptions Profile, Physical Self-Description questionnaire, Objectified Body Consciousness Scale and the Body Appreciation Scale, Body Esteem Scale for Children, Body Appreciation Scale-2 for Children, Objectified Body Consciousness Scale-Youth	Most programmes reported successful outcomes, yet there was no consistent approach to the programme design and delivery. Physical activity and fitness-based programmes were the most frequently used intervention type, followed by critical sociocultural perspectives and programmes focused on movement experiences and body functionality. Whilst fitness-based programmes were generally effective in improving body image and related phenomena, future research should explore the mechanisms associated with these changes and further consider how sociocultural perspectives can be used to support body image programmes.
Pediatric obesity treatment, self-esteem, and body image: A systematic review with meta-analysis	To determine the effect of multicomponent pediatric obesity treatment interventions on self-esteem and body image.	BodyEsteem Scale for Adolescents and Adults, Body Esteem Scale for Children, Body Satisfaction Scale, Children's Body Image Scale, Child Eating Disorder Examination, Eating Disorder Examination Questionnaire, Eating Disorder Inventory, Eating Disorder Inventory, Impact of Weight on Quality of Life, Kids Eating Disorder	meta-analysis of 40 studies (n = 2729) indicated improvements in body image postintervention, maintained at follow-up

**Supplementary Table S2.** Description of objectives, study locations, assessment instruments and main results of systematic reviews on body image in children and adolescents (zero to 19 years of age)			
TITLE	OBJECTIVE	BODY IMAGE ASSESSMENT INSTRUMENTS	KEY RESULT
		Survey, Multidimensional Body-Self Relations Questionnaire, Implication-measuring method designed by Osgood, SelfPerception Profile for Adolescents, Self-Perception Profile for Children, Youth Eating Disorder Examination Questionnaire	
Research Review: Effectiveness of universal eating disorder prevention interventions in improving body image among children: a systematic review and meta-analysis	To examine the effectiveness of universal eating disorder prevention interventions in improving body image, internalization of appearance ideals, and self-esteem among children aged 5–17 years old.	Body Esteem Scale for Children, Developmental assets profile, Rosenberg Self-esteem scale, Body satisfaction scale, eating attitudes test, perception of teasing scale, sociocultural attitudes towards appearance questionnaire 3, Body Esteem Scale for Adolescents and Adults, Self-Perception Profile for Children,	Universal interventions were found to be effective in improving children's body esteem, self-esteem, and internalization of appearance ideals at postintervention and at follow-up timepoints. Subgroup analyses found that girls benefited more from these interventions than boys. Multisessional interventions with an optimal duration of approximately one month were found to be more effective.
Systematic Review of Digital Interventions for Adolescent and Young	To critically evaluate current evidence on the use of digital interventions for adolescent	Body Appreciation Scale, Body Appreciation Scale-2, Body Shape Questionnaire, Body Shape Questionnaire-Short Form, Body-Image Acceptance and Action Questionnaire, Objectified Body Consciousness Scale, Body Esteem Scale for	Nine of 15 interventions were effective in improving at least one body image outcome from pre-post intervention; however, effect sizes were mostly small-medium, and few effects were maintained at follow-up. Studies were

**Supplementary Table S2.** Description of objectives, study locations, assessment instruments and main results of systematic reviews on body image in children and adolescents (zero to 19 years of age)			
TITLE	OBJECTIVE	BODY IMAGE ASSESSMENT INSTRUMENTS	KEY RESULT
Adult Women's Body Image	and young adult women's body image.	adolescents and adults, Body Esteem Scale, Body Image Satisfaction Scale, Body image change inventory, Body Image Importance Scale, Body-self-integration scale, Body Appearance Satisfaction Scale of the Multidimensional Body-Self Relations Questionnaire, Body Complexity Questionnaire, Child Figure Rating Scale, Eating Disorder Examination Questionnaire, Weight concerns subscale, Shape concerns subscale of the EDEQ), Drive for thinness subscale of Eating Disorder Inventory, Body dissatisfaction subscale of Eating Disorder Inventory, Weight Concerns scale, Physical Appearance Comparison Scale, Sociocultural Attitudes Toward Appearance Questionnaire, appearance internalization subscale, media pressures subscale, Sociocultural Attitudes Toward Appearance Questionnaire 4, Visual Analog Scales, Self-objectification Questionnaire, Stunkard Figure Rating Scale, Body dissatisfaction scale	heterogeneous in terms of delivery format, duration, sample characteristics, quality, and outcomes.
School-Based Interventions Improve	Of existing school-based interventions designed to	Not reported	We found a signifcantly larger efect on positive body image and media literacy in the intervention

**Supplementary Table S2.** Description of objectives, study locations, assessment instruments and main results of systematic reviews on body image in children and adolescents (zero to 19 years of age)			
TITLE	OBJECTIVE	BODY IMAGE ASSESSMENT INSTRUMENTS	KEY RESULT
Body Image and Media Literacy in Youth: A Systematic Review and Meta-Analysis	enhance media literacy in order to reduce body dissatisfaction and to promote a positive body image		compared to control groups. However, heterogeneity was substantial for both outcomes. Results suggest that media literacy interventions have the potential to improve media literacy and reduce body dissatisfaction. Interventions that worked with the principle of induction of cognitive dissonance were the most effective.
The effect of cognitive behavior therapy on body image and self-esteem in female adolescents: a systematic review and meta-analysis	to analyze the effects of cognitive-behavior approach on these two variables, we decided to review the effects of cognitive-behavior approach on body image and selfesteem in adolescent girls systematically.	Body–Self Relations Questionnaire, Body Image Concern Inventory, Body Dysmorphic Disorder Modification of the Y-Bocs, Body Appreciation Scale	Metaanalysis results on seven studies (228 participants) showed that the group receiving cognitive-behavior counseling was significantly better than the control group regarding body image. Meta-analysis results of this study represent effectiveness of cognitive-behavior therapy on body image of adolescent girls.
What works in secondary schools? A systematic review of classroom-based body image programs	Identified gap in the literature through a systematic review of studies evaluating body image programs that have been	Body Dissatisfaction Subscale of Eating Disorders Inventory, Body Satisfaction Visual Analogue Scale, Adapted version of the Body Dissatisfaction Subscale of the EDI that is modified to represent the common areas of male body dissatisfaction, Shape	Seven programs improved participants' body image on at least one measure. No programs were effective in improving body image in boys and girls.

**Supplementary Table S2.** Description of objectives, study locations, assessment instruments and main results of systematic reviews on body image in children and adolescents (zero to 19 years of age)			
TITLE	OBJECTIVE	BODY IMAGE ASSESSMENT INSTRUMENTS	KEY RESULT
	conducted with secondary school students in the classroom environment.	Concerns subscale of the Eating Disorder Examination, Weight Concerns subscale of the Eating Disorder Examination, Stunkard Figure rating scale-discrepancy score, Weight Subscale of the Body Esteem Scale for adolescents and young adults, Body Areas Satisfaction Scale of Multidimensional Body Self Relations Questionnaire, Self' Physical Appearance Ratings, Satisfaction with muscles subscale of the Body Satisfaction Subscale of the Body Satisfaction and Body Change Inventory, Satisfaction with weight Subscale of the Body Satisfaction and Body Change Inventory.	
**Sociocultural influences on body image**			
Effects of fitspiration content on body image: a systematic review	To analyze the relationship between individuals exposure to fitspiration content and body image measures or associated	Social media usage questions, Body dissatisfaction Questions, Appearance Anxiety Inventory (AAI), Dissatisfaction subscale from the Eating Disorders Inventory-3, Appearance Comparison Scale, Body Esteem Scale for Adolescents and Adults (BESAA), Physical Appearance Comparison	Twenty articles met the eligibility criteria and were included. Nineteen studies analyzed the relationship between fitspiration and body image, twelve analyzed the association between exposure to fitspiration and physical appearance comparison tendencies, and nine analyzed the association between fitspiration content and mood. One study

**Supplementary Table S2.** Description of objectives, study locations, assessment instruments and main results of systematic reviews on body image in children and adolescents (zero to 19 years of age)			
TITLE	OBJECTIVE	BODY IMAGE ASSESSMENT INSTRUMENTS	KEY RESULT
	variables (e.g., appearance comparison).	Scale-Revised (PACS-R), Sociocultural Attitudes Toward Appearance Questionnaire-4 Revised (SATAQ-4R), Exercise Motivation Inventory-2 (EMI-2), Visual Analogue Scale (VAS), Dissatisfaction subscale from the Eating Disorder Inventory (EDI), Upward Physical Appearance Comparison Scale (UPACS), Body Attitude Test (BAT), State Appearance Comparison Scale (SACS), Physical Appearance Comparison Scale (PACS), Multidimensional Body-Self Relations Questionnaire Appearance Scales (MBSRQ-AS), International-Positive and Negative Affect Scale-Short Form (IPNAS-SF), Body Dissatisfaction (Body Image States Scale (BISS), Body Appreciation Scale—2), Body Change Inventory (BCI), Internalization of the ideal body (Sociocultural Attitudes Towards Appearance Scale-3 (SATAQ-3), Self-Compassion Scale, Revised Male Body Attitudes Scale (MBAS-R)	analyzed the association between frequency of viewing fitspiration content and motives for exercise. Results showed that exposure to "fitspiration" increased individuals' body dissatisfaction, physical appearance comparisons, and negative mood, especially in younger populations.

**Supplementary Table S2.** Description of objectives, study locations, assessment instruments and main results of systematic reviews on body image in children and adolescents (zero to 19 years of age)			
TITLE	OBJECTIVE	BODY IMAGE ASSESSMENT INSTRUMENTS	KEY RESULT
A systematic review on the effects of media disclaimers on young women's body image and mood	To systematically review the available literature on media disclaimers and women's body image and mood	Multidimensional Body-Self Relations, Questionnaire-Appearance, Evaluation Subscale (trait Body dissatisfaction), Visual analogue scale for state, body image and mood, Sociocultural Attitudes Towards Appearance Questionnaire, Physical Appearance, Comparison Scale, Eating Attitudes test, Eating disorder inventory, Experimenter made likert type questions, Visual analogue scales for body dissatisfaction, 3 questions for state appearance comparison, Tobii eye tracking, Internalization-Thin/ Low Body Fat subscale of Sociocultural Attitudes Toward Appearance, questionnaire-4, physical Appearance Comparison Scale-Revised, Drive for thinness scale, Rosenberg Self-Esteem Inventory, Body Area Satisfaction subscale of Multi-Dimensional Body-Self Relation Questionnaire, Likert-type questions to assess model attractiveness, Objectified Body Consciousness Scale, Physical Self-Description Questionnaire (physical self-esteem), Eating Disorder Examination Questionnaire, Visual analogue scales to assess mood and body	Overall, disclaimers were ineffective at reducing women's body dissatisfaction and negative affect following exposure to thin ideal images, and in some cases were actually harmful to women's body image. For women high in trait body dissatisfaction and thin ideal internalization, warning labels increased body dissatisfaction after exposure to thin ideal images. For women high in trait social and appearance comparison specific disclaimers that outlined how the images had been altered resulted in increased body dissatisfaction after exposure to thin ideal images. Therefore, overall, disclaimers were ineffective at ameliorating the negative effects of exposure to thin ideal media. Future research should examine the impact of media disclaimers on the body image of adolescents and men.

**Supplementary Table S2.** Description of objectives, study locations, assessment instruments and main results of systematic reviews on body image in children and adolescents (zero to 19 years of age)			
TITLE	OBJECTIVE	BODY IMAGE ASSESSMENT INSTRUMENTS	KEY RESULT
		dissatisfaction, State Appearance Comparison Scale, Perceived realism scale, tate Appearance Comparison Scale, hin-Low Body Fat subscale of Sociocultural Attitudes Towards Appearance Questionnaire, Objectified Body Consciousness Scale for Preadolescent and Adolescent Youth, Social Comparison to Models and Peers Scale, Rosenberg's self-esteem scale.	
Internalization of body shape ideals and body dissatisfaction: A systematic review and meta-analysis	To summarizes existing evidence on the relationship between the internalization of body shape ideals (IBSI) and body dissatisfaction	Body shape questionnaire, body shape satisfaction questionnaire, body dissatisfaction subscale of the Eating Disorders Inventory, internalization of body shape ideals, male body attitudes scale, multidimensional media influence scale, multidimensional body-self relations questionnaire, sociocultural attitudes towards appearance questionnaire, body parts satisfaction scale, sociocultural internalization of appearance questionnaire adolescents	Results revealed medium to very relationships, all these being largely similar in male and female individuals.
Uso de redes sociales y factores de riesgo para el desarrollo de trastornos	The objective of this systematic review is to collect and analyze scientific	Not reported	Evidence suggests that the use and frequency of use of social networks is significantly associated with

**Supplementary Table S2.** Description of objectives, study locations, assessment instruments and main results of systematic reviews on body image in children and adolescents (zero to 19 years of age)			
TITLE	OBJECTIVE	BODY IMAGE ASSESSMENT INSTRUMENTS	KEY RESULT
relacionados con la alimentación en Espana: una revisión sistemática	evidence on the relationship between the use of networks social and the increase in risk factors for the development of eating disorders in the Spanish population.		body dissatisfaction in adolescents in the Spanish population.
The Role of the Media in Body Image Concerns Among Women: A Meta-Analysis of Experimental and Correlational Studies	Examined experimental and correlational studies testing the links between media exposure to women's body dissatisfaction	Not reported	The findings support the notion that exposure to media images depicting the thin-ideal body is related to body image concerns for women.
Prevalence of body weight dissatisfaction among adolescents: a systematic review	To identify the prevalence of weight dissatisfaction among adolescents aged 10–19 years and stratify the analysis by sex.	Questionnaire, scale	The prevalence of weight dissatisfaction ranged from 18.0 to 56.6% in both sexes.
The Meaning and Factors That Influence the Concept of Body Image: Systematic Review and Meta-	To analyze the meaning of, and factors influencing, the body image construct, from the perspectives of	Semi-structured Interview, Mixed-methods approach using body image assessment instruments	The synthesis revealed six themes: self-perception of body image; opinions of friends and colleagues; opinions of family; specific features of the school environment; expectations perceived across the

**Supplementary Table S2.** Description of objectives, study locations, assessment instruments and main results of systematic reviews on body image in children and adolescents (zero to 19 years of age)			
TITLE	OBJECTIVE	BODY IMAGE ASSESSMENT INSTRUMENTS	KEY RESULT
Ethnography from the Perspectives of Adolescents	adolescents in western countries, through an interpretative systematic review of qualitative studies.		mass-media; and strategies, practices, and self-management of body image. An explanatory model was developed that showed adolescents' development of body image and the path towards its establishment.
**Sociodemographic aspects related to Body Image**			
Normative body image development: A longitudinal meta-analysis of mean-level change	To provide as complete as possible a picture of normative body image development across the lifespan, using meta-analytic techniques to synthesize mean-level change in body image over time, and to model the influences of age, gender, construct, birth cohort, attrition, and time lag.	not reported	Boys and men showed fluctuations in overall body image with net-improvements between ages 10 and 24. Girls and women showed worsening body image between ages 10 and 16, but improvements between ages 16 and 24.

**Supplementary Table S2.** Description of objectives, study locations, assessment instruments and main results of systematic reviews on body image in children and adolescents (zero to 19 years of age)			
TITLE	OBJECTIVE	BODY IMAGE ASSESSMENT INSTRUMENTS	KEY RESULT
Percepção da imagem corporal e nível socioeconômico em adolescentes: revisão sistemática	To discuss the relationships between body image perceptions and socioeconomic factors such as income, level of education, and ethnicity among adolescents	not reported	The main findings are: North-American lower class youths present greater chances for obesity, but this is not true in Brazil; girls and boys have different behaviors concerning body image perceptions, despite ethnicity or socioeconomic status; Caucasian girls show more dissatisfaction with their body images and greater search for diets than African American girls, who seem to suffer less influence of the current beauty patterns; youth of lower socioeconomic status presents a tendency to desire larger bodies.
Body image perception of African immigrants in Europe	To evaluate body dissatisfaction and weight discrepancy among African immigrants in Europe compared to people still living in Africa, tracing a pattern of geographical variation where possible	7 silhouettes, 9 silhouettes, Self-administered Questionnaire, 9 silhouettes, Body Shape Questionnaire, Body Image Concern (6 hotographic silhouettes, 9 silhouettes, 10 silhouettes), self-administered questionnaire, 18 silhouettes, Photographic Figure Rating Scale, Body Appreciation Scale, 50 high-resolution photographic images (self-administered questionnaire), 8 silhouettes developed, Multidimensional Body-Self Relations Questionnaire.	Among African residents, the body preferences depend on the country of residence and their socio-cultural status. Ethnic groups living in great isolation or with low incomes still have an ancestral idea of beauty, preferring a shapely body. However ethnic groups living in urban areas are moving toward. Both residents and migrants are at high risk of nutritional disorders due to the adoption of Western beauty ideals. The results suggest that body dissatisfaction and BMI are increasing from

**Supplementary Table S2.** Description of objectives, study locations, assessment instruments and main results of systematic reviews on body image in children and adolescents (zero to 19 years of age)			
TITLE	OBJECTIVE	BODY IMAGE ASSESSMENT INSTRUMENTS	KEY RESULT
			Southern Africa to Europe according to a geographical gradient. Westernization of beauty ideals, preferring underweight or normal weight bodies.
Ethnicity and Body Dissatisfaction Among Women in the United States: A Meta-Analysis	To conduct a meta-analysis to investigate the relationship between ethnicity and body dissatisfaction among women in the United States	Body Cathexis Scale, Body-Esteem Scale, Body Areas Satisfaction Scale, Multidimensional Body-Self Relations Questionnaire, Eating Disorder Inventory - EDI, Eating Disorder Inventory, Stunkard Figure Rating Scale, Body Shape Questionnaire, Body-Image Ideals Questionnaire, General weight concern, General body dissatisfaction, Figure Rating Scale, Body Disapproval subscale of the Million Adolescent Clinical Inventory, Body–Self Relations Questionnaire, Body Image subscale of the Self-Image Questionnaire for Young Adolescents	The average d for the White–Black comparison was 0.29, indicating that White women are more dissatisfied, but the difference is small. All other comparisons were smaller, and many were close to zero. The findings directly challenge the belief that there are large differences in dissatisfaction between White and all non-White women and suggest that body dissatisfaction may not be the golden girl problem promoted in the literature
A systematic review of body dissatisfaction and sociocultural messages	To systematically review the literature related to body dissatisfaction among preschool children, and the factors that are associated	Collins' Figure Drawing; Body Esteem Scale (BES) and Weight Concerns Scale; Brief version of BES and CFD; children's Figure Rating Scale; Appearance satisfaction scale developed for study; Digital personalized figure rating task Child	Children under the age of 6 years old experience body dissatisfaction, however, the proportion of children who are dissatisfied varied from around 20% to 70%, depending on the method of assessment. The literature was divided on whether

**Supplementary Table S2.** Description of objectives, study locations, assessment instruments and main results of systematic reviews on body image in children and adolescents (zero to 19 years of age)			
TITLE	OBJECTIVE	BODY IMAGE ASSESSMENT INSTRUMENTS	KEY RESULT
related to the body among preschool children	with the development of body image dissatisfaction	interview – weight concerns and appearance related question, CFD adapted for Chinese children, Silhouette figure selection, Children observed in playroom, Nine body silhouettes modified based on Tiggemann and Pennington.	preschool aged girls experience more body dissatisfaction than boys. Parental influence appears to be an important factors in the development of preschool children's body dissatisfaction and attitudes. However, more research is needed to understand the influences of children's peers and the media.
Perception of body size and dissatisfaction in children aged 3 to 6: a systematic review	The aim of this study was, therefore, to analyse the most significant findings on body dissatisfaction and body-size perception among children aged 3 to 6 (second cycle of pre-school education).	Collins Figure Drawing, Tiggemann and Pennington's Silhouette scale, Three silhouette method, Children's body size attitudes scale, body esteem scale (BES), Weight Concems Scale, Doors of diffening size, Body figures to which were added the girls faces	With regard to dissatisfaction, the analysis shows that it very prevalent at these ages. Prevalence varies significantly across the research studies, however, with percentages ranging between 9% and 84%. The study of body image in the second cycle of pre-school yields varying.
**Physical Activity and Body Image**			
Physical activity and body image among men and boys: A meta-analysis	Update to the literature regarding the relationship between physical activity and body image	Children and Youth Physical Self-perception Profile: Body Attractiveness and Physical Self-worth, Tennessee Self-Concept Scale, State Self-Esteem Scale Appearance active non-athletes Subscale, Drive for Muscularity, Body Image	The results of this meta-analysis support the notion that physical activity is positively associated with body image among men. The relationship seems robust among men and the overall effect size was medium which is somewhat consistent with

**Supplementary Table S2.** Description of objectives, study locations, assessment instruments and main results of systematic reviews on body image in children and adolescents (zero to 19 years of age)			
TITLE	OBJECTIVE	BODY IMAGE ASSESSMENT INSTRUMENTS	KEY RESULT
		Satisfaction questionnaire, Body Cathexis Scale, Body Esteem Scale for Children, Self-Perception Profile for Adolescents, perception of body weight, perception of body appearance, Perceptions of Body Attractiveness and Satisfaction, Contour Drawing Scale, Multidimensional Body-Self Relations Questionnaire, : Social Physique Anxiety Scale, Body Dissatisfaction Index, Body Shape Questionnaire, Body Dissatisfaction via silhouette matching technique	previous meta-analyses, which have found small to medium sized relationships between physical activity and body image for men.
Effects of exercise motivations on body image and eating habits/behaviours: A systematic review	To summarise all the available scientific evidence, without restrictions on study design, exploring the effects or associations between exercise motivations and (a) body image or (b) eating habits/behaviours in adult exercisers.	Social physique anxiety scale, eating disorder examination questionnaire, multidimensional self-esteem inventor, weight influenced self-esteem questionnaire, body appreciation scale, body surveillance - objective body consciousness scale, functional satisfaction - embodied image scale	Autonomous motivations (eg, for pleasure, health, wellbeing) and health-focused exercise were associated with positive body image and healthy eating habits/behaviours, where as exercising for ppearance-related and other controlled reasons was inversely related to both outcomes.

**Supplementary Table S2.** Description of objectives, study locations, assessment instruments and main results of systematic reviews on body image in children and adolescents (zero to 19 years of age)			
TITLE	OBJECTIVE	BODY IMAGE ASSESSMENT INSTRUMENTS	KEY RESULT
Body image of healthy adolescent women and its association with physical activity: a systematic review	To evaluate the related scientific production on body image in healthy female adolescents and its association with physical activity. healthy and its association with physical activity	Body shame scale, Embodied Image Scale, Body Image Satisfaction Scale, Body attitudes questionnaire, Questionnaires, Bodydissatisfaction, Feelings and attitudes towards the body scale, Physical appearance, interview based in Buddeberg-Fischer questionnaire, Body image subscale, Figure rating scale, Children and Youth's Physical Self-Perception Profile, Body areas satisfaction scale, Contour Drawing Rating scale knowledge inventory, a photo with five different silhouettes	The most published paper concentrations were found in Europe (61.2 %). Several studies (95%) used questionnaires as a research instrument to assess physical activity and 55% studies used scales to analyze the body image. No increase was found in the production of literature involving body image study and physical active behavior in healthy female adolescents. In conclusion, the main findings of this review relate to the lack of parameters for the measurement of physical activity and body image analysis. Thus, although it is a current thematic area in the literature, it is possible to consider that the diversity of methodological design between studies makes it difficult to compare the data, making it imperative to standardize instruments and definitions
Body Image and its Role in Physical Activity: A Systematic Review	To investigate the associations between body image and perceived physical competence and sports attrition in children.	not reported	Evidence revealed that children younger than 7 years have an inflated self-perception and are eager to participate in activities regardless of competence. Between 7 to 10 years of age, children begin to more accurately perceive their skills and draw

**Supplementary Table S2.** Description of objectives, study locations, assessment instruments and main results of systematic reviews on body image in children and adolescents (zero to 19 years of age)			
TITLE	OBJECTIVE	BODY IMAGE ASSESSMENT INSTRUMENTS	KEY RESULT
			comparisons with their peers. Elevated body mass index (BMI) becomes a deterrent between 6 to 11 years. After 12 years, teasing and gender identification issues become causes of attrition. In adolescence, body image becomes a significant determinant of continuation of physical activity, more than actual skill. Perceived physical competency and body image do not appear to affect sports attrition in children younger than 7 years.
Physical Activity and Body Image Perception in Adolescents: A Systematic Review	I. To evaluate the associations between body image or body image dissatisfaction and physical activity according to body mass index or weight status in adolescents; II. to allow a more complete and up-to-date synthesis of the studies that have been carried out in this area by figural scales over the past decade; III. to extend the analysis on body	Stunkard Rating Scale, Collins for preadolescents, Contour Drawing Rating Scale proposed by Thompson and Gray, silhouettes developed by Kakeshita, BIDA questionnaire, the Body Silhouette Chart developed by Sanchez-Villegas, silhouettes developed by Gardner	The main finding of interest that emerged from most of the 28 included studies is the negative association between body image dissatisfaction and physical activity during adolescence: as physical activity increases, body image dissatisfaction decreases.

**Supplementary Table S2.** Description of objectives, study locations, assessment instruments and main results of systematic reviews on body image in children and adolescents (zero to 19 years of age)			
TITLE	OBJECTIVE	BODY IMAGE ASSESSMENT INSTRUMENTS	KEY RESULT
	image or body image dissatisfaction to physical activity contexts related to lifestyle -not just structured physical activity.		
The Influence of Dance Practice on the Body Image of Adolescents: A Systematic Review	To systematically review the influence of dance practice on the body image of adolescents.	Questionnaire, Body Attitude Questionnaire with 44 items, Children and Youth Physical Self- Perception Profile with 36 items, Likert Scales Assessment with 10 items, Stunkard's, Body Shape Questionnaire, Contour Drawing Rating Scale (CDRS) with 9 silhouettes	Older dancers had better perception of body image when compared to the young subjects. However, female dancers with a more experience were more unsatisfied with the body image than the less experienced. The practice of dancing improved the body image perception, though this positive perception may be influenced by clothing, body mass index, hip, and waist.
**Personality and Cognitive Thinking to understand Body Image**			
Personality and body image: A systematic review	reviewed the evidence for personality as a correlate of body image	not reported	The results indicated that negative body image was associated with higher levels of Neuroticism and lower levels of Extraversion
Cognitive biases to appearance-related stimuli	To examine the relationship between cognitive processing	Negative Physical Self Scale-Fatness, Body Checking Questionnaire, Derriford Appearance	The extant literature provides robust support for the presence of attention biases toward body image-

**Supplementary Table S2.** Description of objectives, study locations, assessment instruments and main results of systematic reviews on body image in children and adolescents (zero to 19 years of age)			
TITLE	OBJECTIVE	BODY IMAGE ASSESSMENT INSTRUMENTS	KEY RESULT
in body dissatisfaction: A systematic review	of body image-related stimuli and body dissatisfaction	Scale, Physical Appearance State and Trait Anxiety Scale, Appearance Schemas Inventory- Revised.	related stimuli among individuals with high levels of body dissatisfaction compared to those with lower levels of concerns. Evidence was also found for the existence of judgment biases and memory biases. Furthermore, results suggest that body image-related cognitive biases, and levels of body dissatisfaction can be manipulated. Initial evidence was also found for differential patterns of biases toward “fat” and “thin” stimuli
**Studies on Body Image with Specific Populations**			
Association between verweight/obesity with depression, anxiety, low self-esteem, and body dissatisfaction in children and adolescents: a systematic review and meta-analysis of observational studies	To summarize published articles on the association between overweight/obesity with risk of depression, anxiety, low self-esteem, and body dissatisfaction among children and adolescents.	Harter’s self-perceptions profile, Harter’s self-perceptions profile for Children.	A positive significant association was found between overweight and obesity with risk of low selfesteem, respectively. A significant positive association was found between obesity and risk of body dissatisfaction. Moreover, no association was found between overweight and risk of body dissatisfaction among children and adolescents. Also, no association was observed between overweight/obesity and risk of depression and anxiety.

**Supplementary Table S2.** Description of objectives, study locations, assessment instruments and main results of systematic reviews on body image in children and adolescents (zero to 19 years of age)			
TITLE	OBJECTIVE	BODY IMAGE ASSESSMENT INSTRUMENTS	KEY RESULT
Body Image in Adolescent Pregnancy	To review the existing literature on body image in adolescent pregnancy and explore concepts about the relationship between the two.	Interviews, physical exam mental status exam, Edinburgh postnatal depression scale; Parenting stress index (3rd ed.), eating disorder inventory. Theme from focus group analyzed, Tennessee self concept scale questionnaire, and diary entry analysis of self-image and body image.	The search yielded a total of 149 studies, of which 6 were relevant to the specific topic and age group. The very limited research shows a dichotomy in body image perception during pregnancy in adolescence; some studies show an increase in body image disturbance and dissatisfaction during pregnancy in adolescents, and other studies reviewed found that the majority of pregnant adolescents had positive body image and positive attitudes towards weight gain. A bidirectional link between depression and negative body image in adolescent pregnancy is suggested.
Body image in children and adolescents diagnosed with the human immunodeficiency virus: a systematic review	To estimate the prevalence of body image dissatisfaction, to describe the assessment methods, and to identify associated factors in children and adolescents diagnosed with HIV	Dusek's short form of the Secord-Jourard Body Cathexis Scale; Silhouette Scale previously validated with adolescents from Florianópolis; Silhouette scale validated by Kakeshita et al.	Prevalence of body image dissatisfaction due to thinness was between 36.7–52.0% in males and 28.1–36.4% in females, and body image dissatisfaction due to overweight was between 8.0–31.2% in males and 21.9–50.0% in females. Factors associated with body image dissatisfaction were as follows: female sex, older age, low levels of physical activity, low self-esteem, higher body fat, higher body weight, greater arm muscle area,

**Supplementary Table S2.** Description of objectives, study locations, assessment instruments and main results of systematic reviews on body image in children and adolescents (zero to 19 years of age)			
TITLE	OBJECTIVE	BODY IMAGE ASSESSMENT INSTRUMENTS	KEY RESULT
			triceps skinfold thickness, and higher body mass index.
Body image of children and adolescents with cancer: A metasynthesis on qualitative research findings	To synthesize the existing qualitative findings in order to facilitate understanding of the body image experience of children and adolescents with cancer.	Semi structured interview, individual interview, focus group, and participant observation, online survey, telephone interviews and focus group.	Children and adolescents with cancer also experience various problems associated with changes in their body image. Repeated courses of treatment lead to loss of a normal, orderly life, and might even result in changes in interpersonal interactions. In response to body image change, individuals with cancer develop self-protective, coping strategies. Children and adolescents who experience life-threatening cancer come to face body image change positively, and might hold a confident attitude toward their future.
Body image of children and adolescents with cancer: A systematic review	To identify (1) differences in body image (BI) between children and adolescents with cancer and healthy controls; (2) relationships between BI and demographic or medical variables; (3) implications of BI for	Piers-Harris Self-Concept Scalea. Physical and visible impairment rating scaleb, Sentence completion test, Quality of Life Questionnaire for Children and Adolescents with Cancer, the German instrument (PEDQOL), Self-Perception Profile for Childrenc, Semi-structurured interview, Body Image Instrument (BII), Problem list, Survey instrument, Offer Self-Image Questionnaire for	There was no consistent evidence regarding body image differences between children and adolescents with cancer and healthy controls. Relations between body image and gender, disease characteristics, and psychological adjustment were found. Changes in body image have adverse implications for self-esteem and adjustment, but can be moderated by social support. Future research should also adopt

**Supplementary Table S2.** Description of objectives, study locations, assessment instruments and main results of systematic reviews on body image in children and adolescents (zero to 19 years of age)			
TITLE	OBJECTIVE	BODY IMAGE ASSESSMENT INSTRUMENTS	KEY RESULT
	psychological adjustment; and (4) relationship between BI<br>and social support.	Adolescents (OSIQ), Medical Outcome Study Scale (MOS-24), Esteem Scale, Worries questionnaire, Self-Perception Profile for Adolescent, Personality Inventory (MAPI), Telephone interview, Behavioral Assessment System for Children (BASC), Kinetic family Drawing-Revised (KFD-R), Physical and visible impairment rating scaleb, Self-Image Questionnaire for Young Adolescents (SIQYA), Body Cathexis Scale (BCS), Body Image Avoidant Questionnaire (BIAQ), Situational Inventory of Body Image Distress (SIBID), Self-developed questionnaire	broader definitions of body image, and cancer-specific age-appropriate body image measures are needed to increase sensitivity of this work.
Body image of children and adolescents with chronic illness: A meta-analytic comparison with healthy peers	To integrate the available research across a broad range of diseases.	Physical Appearance scale of the SelfPerception Profile for Children or Adolescents, Physical Appearance scale of the Piers-Harris Self-Concept Questionnaire, Physical Self scale of the Offer Self-Image Questionnaire, Draw a Person, Body Image Scale of the “I think I am” questionnaire, Physical Self scale of the Tennessee Self-Concept Scale, Body Esteem Scale, Related scale of the Impact of	Young people with a chronic illness had a less positive body image than their healthy peers although the average size of differences was small. A comparison of diseases showed that young people with obesity, cystic fibrosis, scoliosis, asthma, growth hormone deficits, spina bifida, cancer, and diabetes. Evaluated their body less positively than their healthy peers. Furthermore, levels of body dissatisfaction varied by age at onset

**Supplementary Table S2.** Description of objectives, study locations, assessment instruments and main results of systematic reviews on body image in children and adolescents (zero to 19 years of age)			
TITLE	OBJECTIVE	BODY IMAGE ASSESSMENT INSTRUMENTS	KEY RESULT
		Weight on Quality of Life, Body Image Scale of the Pediatric Quality of Life Questionnaire.	of the disease, method for assessing body image, ethnicity, year of publication, and comparison group. Recommendations are stated for reducing effects of chronic illness on the body image of people with chronic illness.
Body Image Problems in Individuals with Type 1 Diabetes (T1D): A Review of the Literature	To systematically evaluate the empirical evidence concerning body image in individuals with T1D in order to provide an overview of the existing literature	Body Mass Index Silhouette Matching Test (BMI-SMT), 2 items evaluating current body weight/shape concerns developed for this study, SCOFF assessing satisfaction with body weight, Self-perception and Body Awareness subscale, Essen Resource Inventory for Children and Adolescents (ERI-KJ), Monitoring Individual Needs in Diabetes Youth Questionnaire (MY-Q), MIND Youth Questionnaire (MY-Q), Eating Disorder Inventory (EDI), Offer Self-Image Questionnaire (OSIQ), Eating Disorder Examination-Questionnaire (EDE-Q), Contour Drawing Rating Scale (CDR), Items formed a 20-item self-report questionnaire developed specifically for this research to evaluate Type 1 diabetes participants' perceptions about body image, Rorschach items,	Youth with T1D, body dissatisfaction was common and that body concerns were generally greater in youth with T1D than in controls; nine studies did not find any differences in body image problems between participants with and without T1D; three studies described higher body satisfaction in youth with diabetes than in controls; and three studies reported mixed results. Body concerns in individuals with T1D were often found to be associated with negative medical and psychological functioning. The variability and limits in assessment tools across studies, the overrepresentation of female subjects, and the fact that most research in this field is based on cross-sectional data are stressed in the interpretation of these mixed findings.

Supplementary Table S2. Description of objectives, study locations, assessment instruments and main results of systematic reviews on body image in children and adolescents (zero to 19 years of age)

TITLE	OBJECTIVE	BODY IMAGE ASSESSMENT INSTRUMENTS	KEY RESULT
		Appearance Schemas Inventor, The Narcissism Inventory–90, Frankfurter Scales of Body Concept (FKKS), Body Esteem Scale for Adolescents and Adults (BESAA), Shape and weight concern subscales from (Children and Adult) Eating Disorder Examination, assessing shape and weight concerns Items from SelfPerception Profile for Adolescents, assessing self-concept, Body Image Scale (BIS), Body Image Assessment (BIA), Eating Disorders Inventory-3 (EDI-3), Stunkard figure, Diabetes Adjustment Scale (DAS), Eating Disorder Inventory for children (EDI-C), Items assessing body image concerns from drawings tests (drawing of opposite sex person, House-tree-person/HTP) and Rorschach items, Collins's body image silhouette chart, Truby and Paxton's Children's Body Image Scale, Sociocultural Attitudes Towards Appearance Questionnaire-4R (SATAQ-4R), Child Eating Disorder Examination (cEDE), Early Eating Disorder Signs (SEEDS), Body Image States Scale (BISS), Body Image Disturbance Questionnaire (BIDQ), Appearance Schemas Inventory, Body	

**Supplementary Table S2.** Description of objectives, study locations, assessment instruments and main results of systematic reviews on body image in children and adolescents (zero to 19 years of age)			
TITLE	OBJECTIVE	BODY IMAGE ASSESSMENT INSTRUMENTS	KEY RESULT
		Image Ideals Questionnaire (BIQ), 5-point body-satisfaction scale.	
A systematic review examining the association between female body image and the intention, initiation and duration of postpartum infant feeding methods (breastfeeding vs bottle-feeding)	To examine first-time mothers' intention, maintenance and duration in infant feeding behaviour (breastfeeding vs non-breastfeeding/bottle-feeding) and the influence of body image as the deciding variable, specifically looking at the degree of body image as the influence in the intended infant feeding behaviour, and whether the degree of body image influences the maintenance and duration of the intended infant feeding behaviour.	Subscales: shape and weight concern, Body Attitude Questionnaire, body areas satisfaction	Findings suggest that exclusive breastfeeding is more likely in pregnant women with a higher body image, while those with body concerns had less intention to breastfeed or initiate, with those who start having a shorter duration.
Self-concept and body image of people living	To summarize existing evidence regarding body	Questionnaire, scale (not reported)	Results indicate that changes in the body image of people with lupus and their perception are issues

**Supplementary Table S2.** Description of objectives, study locations, assessment instruments and main results of systematic reviews on body image in children and adolescents (zero to 19 years of age)			
TITLE	OBJECTIVE	BODY IMAGE ASSESSMENT INSTRUMENTS	KEY RESULT
with lupus: A systematic review	image in patients with systemic lupus erythematosus, with the following considerations: (a) the perceptions patients have of their body changes; (b) how patients cope with changes in their body; (c) and what their perceptions are of body changes		that must be treated as characteristics of the disease; therefore, they need to receive the same attention as is given to physical disabilities and pain.
Women's experiences of their pregnancy and postpartum body image: a systematic review and meta-synthesis	To review and explore studies of women's perceptions of their body image during pregnancy and postpartum, and to highlight the clinical implications for health professionals working in obstetrics.	Not reported	Three themes were highlighted: "Public Event: 'Fatness' vs. Pregnancy", "Control: Nature vs. Self", and "Role: Woman vs. Mother". Women perceived the pregnant body to be out of their control and as transgressing the socially constructed ideal, against which they tried to protect their body image satisfaction. Women perceived the physical manifestation of the mothering role as incongruent to their other roles as a wife or partner, or working woman. Body dissatisfaction dominated the postpartum period.

Supplementary Table S3. Assessment of the risk of bias of the articles included in the review.

Author (year)	Q1	Q2	Q3	Q4	Q5	Q6	Q7	Q8	Total Score *
**Body Image Measurement Instruments**									
1 Cortês et al. (2013)	1	1	1	1	0	1	1	1	7
**Programs and Interventions focusing on Body Image**									
2 Guest (2020)	0	1	1	0	1	1	NA	NA	6
3 Zuair and Sopory (2020)	0	1	0	0	1	1	1	1	5
4 Kerner et al. (2022)	1	1	1	1	0	1	NA	NA	7
5 Gow et al. (2020)	1	1	1	1	1	1	1	1	8
6 Chua et al. (2013)	1	1	1	1	1	1	1	1	8
7 Mahon and Seekis (2022)	1	1	1	1	1	1	NA	NA	8
8 Kurz et al. (2022)	1	1	1	1	0	1	1	1	7
9 Zamiri-Miandoab et al. (2021)	1	1	1	1	0	1	1	1	7
10 Yager et al. (2013)	1	1	1	1	0	1	NA	NA	7
**Sociocultural influences on Body Image**									
11 McComb and Mills (2020)	0	1	1	0	1	1	NA	NA	6
12 Jerónimo and Carraça (2022)	1	1	1	1	1	1	NA	NA	8
13 Paterna et al. (2021)	0	1	1	1	1	1	1	1	7
14 Marín et al. (2023)	1	1	1	1	1	1	NA	NA	8
15 Grabe et al. (2008)	1	1	1	1	0	1	1	1	7
16 San Martini et al. (2023)	1	1	1	1	0	1	NA	NA	7
17 Tort-Nasarre et al. (2021)	1	1	1	1	1	1	1	0	7
**Sociodemographic aspects related to Body Image**									
18 Pereira et al. (2011)	1	1	1	1	0	1	NA	NA	7
19 Lacroix et al. (2023)	1	1	1	1	0	1	1	1	7
20 Toselli et al. (2016)	1	1	1	1	0	1	0	1	6
21 Grabe and Hyde (2006)	0	1	1	0	0	1	0	1	4
22 Tatangelo et al. (2016)	1	1	1	0	0	1	NA	NA	6
23 León et al (2018)	1	1	1	1	0	1	NA	NA	7
**Physical Activity and Body Image**									
Panão and Carraça (2019)	1	1	1	1	1	1	NA	NA	8
Bassett-Gunter et al. (2018)	1	1	1	1	0	1	1	1	7

Supplementary Table S3. Assessment of the risk of bias of the articles included in the review.

Author (year)	Q1	Q2	Q3	Q4	Q5	Q6	Q7	Q8	Total Score *
Davelaar (2021)	1	1	0	0	0	1	NA	NA	5
Rica et al. (2018)	0	1	1	0	0	1	NA	NA	5
Gualdi-Russo et al. (2022)	1	1	1	1	1	1	NA	NA	8
Monteiro et al. (2018)	1	1	1	1	0	1	NA	NA	7
**Personality and Cognitive Thinking to understand Body Image**									
Allen et al. (2016)	1	1	1	0	0	1	NA	NA	6
Rodgers and DuBois (2016)	0	1	1	1	1	1	NA	NA	7
**Studies on Body Image with Specific Populations**									
Moradi et al. (2022)	0	1	1	1	1	1	1	1	7
Zaltzman et al. (2015)	0	1	0	1	0	1	NA	NA	5
Zanlorenci et al. (2023)	1	1	1	1	1	1	NA	NA	8
Lee et al. (2012)	1	1	1	1	1	1	NA	NA	8
Fan and Eiser (2009)	0	1	1	1	0	1	NA	NA	6
Pinquart (2013)	1	1	1	1	1	1	1	1	8
Troncone et al. (2021)	1	1	1	1	0	1	NA	NA	7
Morley-Hewitt et al. (2020)	1	1	1	1	0	1	1	NA	7
Rodrigues et al. (2021)	1	1	1	1	1	1	NA	NA	8
Hodgkinson et al. (2014)	1	1	1	1	1	1	1	0	7

Q: question; NA: not applicable – Q1: Is the review based on a focused question that is adequately formulated and described?; Q2. Were eligibility criteria for included and excluded studies predefined and specified?; Q3. Did the literature search strategy use a comprehensive, systematic approach?; Q4. Were titles, abstracts, and full-text articles dually and independently reviewed for inclusion and exclusion to minimize bias?; Q5. Was the quality of each included study rated independently by two or more reviewers using a standard method to appraise its internal validity?; Q6. Were the included studies listed along with important characteristics and results of each study?; Q7. Was publication bias assessed?; Q8. Was heterogeneity assessed? *result of the sum of the questions that were answered 1 (yes) and not applicable subtracted from the questions that were answered no (zero).
